# Supplementary material for: Four SpsP neurons are an integrating sleep regulation hub in Drosophila
Source: Sci Adv. 2024 Nov 22;10(47):eads0652. doi: 10.1126/sciadv.ads0652 (PMC11584021; doi:10.1126/sciadv.ads0652)
Supplement: 20241122-1 [file sciadv.ads0652.v1.pdf]

NEUROSCIENCE

Four SpsP neurons are an integrating sleep regulation hub in *Drosophila*

Xihuimin Dai[1], Jasmine Quynh Le[1], Dingbang Ma[1,2], Michael Rosbash[1]*

Copyright © 2024 The Authors, some rights reserved; exclusive licensee American Association for the Advancement of Science. No claim to original U.S. Government Works. Distributed under a Creative Commons Attribution License 4.0 (CC BY).

Sleep is essential and highly conserved, yet its regulatory mechanisms remain largely unknown. To identify sleep drive neurons, we imaged *Drosophila* brains with calcium-modulated photoactivatable ratiometric integrator (CaMPARI). The results indicate that the activity of the protocerebral bridge (PB) correlates with sleep drive. We further identified a key three-layer PB circuit, EPG-SpsP-PEcG, in which the four SpsP neurons in the PB respond to ellipsoid body (EB) signals from EPG neurons and send signals back to the EB through PEcG neurons. This circuit is strengthened by sleep deprivation, indicating a plasticity response to sleep drive. SpsP neurons also receive inputs from the sensorimotor brain region, suggesting that they may encode sleep drive by integrating sensorimotor and navigation cues. Together, our experiments show that the four SpsP neurons and their sleep regulatory circuit play an important and dynamic role in sleep regulation.

INTRODUCTION

Sleep is important for multiple biological processes, which include but are not limited to immune responses, learning and memory, and waste clearance (*1*). While sleep is often defined by behavioral changes such as prolonged immobility, reduced responsiveness to external stimuli, and sleep recovery after deprivation (*2*, *3*), sleep stages and wakefulness are traditionally distinguished by the electrophysiological state of the brain. Mammalian sleep stages and wakefulness are characterized by distinct electroencephalographic patterns, and different sleep stages have even been identified in *Drosophila*, in this case through local field potential recordings (*4*–*7*) and hidden Markov modeling of sleep behavior (*8*). This concordance suggests that sleep is regulated by discrete brain neural circuits in flies as well as mammals and suggests that some fly neurons should be more active when the animal needs to sleep or needs to maintain sleep as has been shown in mammals (*9*).

This search for sleep-relevant neurons overlaps with a basic principle of sleep regulation, namely, the importance of a circadian as well as a homeostasis process (*10*). The circadian process is controlled by the ~150 circadian clock neurons in *Drosophila* (*11*), but fly "sleep centers" remain poorly defined, especially those that change with sleep state, deep versus light sleep for example, or brain regions that are critical for sleep homeostasis.

Recent studies have suggested that the central complex, especially its dorsal fan-shaped body (dFB) and ellipsoid body (EB) neurons, are important for the regulation of sleep and sleep homeostasis. Manipulation of drivers labeling the dFB neurons or the EB neurons caused changes in sleep or sleep homeostasis, and functional studies proposed a recurrent circuit between the dFB neurons, the helicon cells, and the EB neurons, which incorporates signals from the circadian neurons (*12*, *13*). Nonetheless, these studies have been constrained by the modest availability and specificity of individual Gal4 drivers and have limited insights into dynamic neuronal activity patterns across the fly brain during natural changes in sleep drive. On the other hand, a recent study has revealed that distinct neuronal ensembles are active during different sleep states (*14*), but the identities and projection patterns of these active neurons remain unknown.

To overcome this limitation of the Gal4 system and search for neurons that respond to sleep drive change in a natural context, we instead began by using calcium-modulated photoactivatable ratiometric integrator (CaMPARI) to take an irreversible snapshot of whole-brain calcium activity in sleep-deprived flies. The results showed that the protocerebral bridge (PB), another component of the central complex, exhibits a substantially higher calcium activity in flies with high sleep drive than any other easily detectable brain location. Following a thermogenetic activation screen with sparsely labeled split Gal4 drivers, we identified three sleep-promoting subsets of PB neurons, namely, four SpsP neurons, ~20 pairs of EPG neurons, and ~9 pairs of PEcG neurons. The four SpsP neurons alone are strongly sleep promoting and show consistent inhibition as well as activation sleep phenotypes. Through connectivity assays, we further show that they serve as a sleep regulation hub: Each SpsP neuron innervates all the PB glomeruli in the hemi-brain, receiving signals from and sending signals back to EB through EPG columnar neurons and PEcG columnar neurons, respectively, as well as by integrating dopaminergic signals.

RESULTS

Calcium activity of PB increases with sleep deprivation

To address cellular mechanisms of sleep drive in a natural context, we took an unbiased snapshot of whole-brain calcium levels by pan-neuronally expressing CaMPARI. It is a photoconvertible calcium sensor, which irreversibly converts green fluorescent protein (GFP) to red fluorescent protein (RFP) in the presence of calcium and ultraviolet (UV) light (*15*, *16*). CaMPARI imaging has been validated and used in several studies to indicate the neuronal activity responses in flies to behaviors including olfaction (*17*), bitter taste processing (*18*), and hedonic hunger (*19*). The RFP/GFP ratio after UV exposure is an indicator of relative calcium levels and, therefore, serves as a proxy for neuronal activity.

Flies exhibit rebound sleep after sleep deprivation (*2*, *3*), supporting the notion that the sleep drive of an animal increases with wakefulness and dissipates with sleep. To identify the neurons that respond to high sleep drive, we carried out ex vivo imaging with

[1]Howard Hughes Medical Institute, Brandeis University, Waltham MA 02454, USA.
[2]Interdisciplinary Research Center on Biology and Chemistry, Shanghai Institute of Organic Chemistry, Chinese Academy of Sciences, Shanghai, China.
*Corresponding author. Email: rosbash@brandeis.edu

pan-neuronally expressed CaMPARI at Zeitgeber time (ZT)1, i.e., 1 hour after lights on in a 12-hour:12-hour light:dark cycle. This was done with two groups of flies: flies with high sleep drive that had been sleep deprived for 13 hours, i.e., for the whole night +1 hour from ZT12 to ZT1 (SD group); and flies harboring relatively low sleep drive, which had slept undisturbed for the whole night and also assayed at the same time of day (CTRL group) (Fig. 1A). While whole-brain CaMPARI imaging successfully labels neuropils throughout the entire brain, photoconversion within deep brain regions such as the EB and the FB might be limited by the penetration capability of the UV light and the laser for confocal imaging. We found that the signal-to-noise ratios are weaker in neuropils deep in the brain compared to neuropils on the brain surface. We therefore normalized the CaMPARI signal of each SD neuropil to the same neuropil of the control group (see Materials and Methods for details). Among all the neuropils examined, the increase of CaMPARI signal after sleep deprivation in the PB was the most robust and reliable (Fig. 1B and fig. S1D). The signal in the EB, a brain region that has been reported to encode sleep homeostasis (*20*), also increased with sleep deprivation. There was little change in signal in the antenna lobe (AL) and the optic lobe (fig. S1D).

To further address the extent to which increased PB calcium after sleep deprivation is caused by increased sleep drive, we also carried out ex vivo CaMPARI imaging at ZT3 after 2 hours of recovery sleep (Fig. 1A). After 13 hours of sleep deprivation, flies in the SD group showed significantly higher PB calcium activity than the control group at ZT1 (Fig. 1C), and after an additional 2 hours of recovery sleep at ZT3, there was no statistically significant difference in the calcium activity of the PB between the SD group and the CTRL group (Fig. 1D). To further confirm the calcium activity of increase

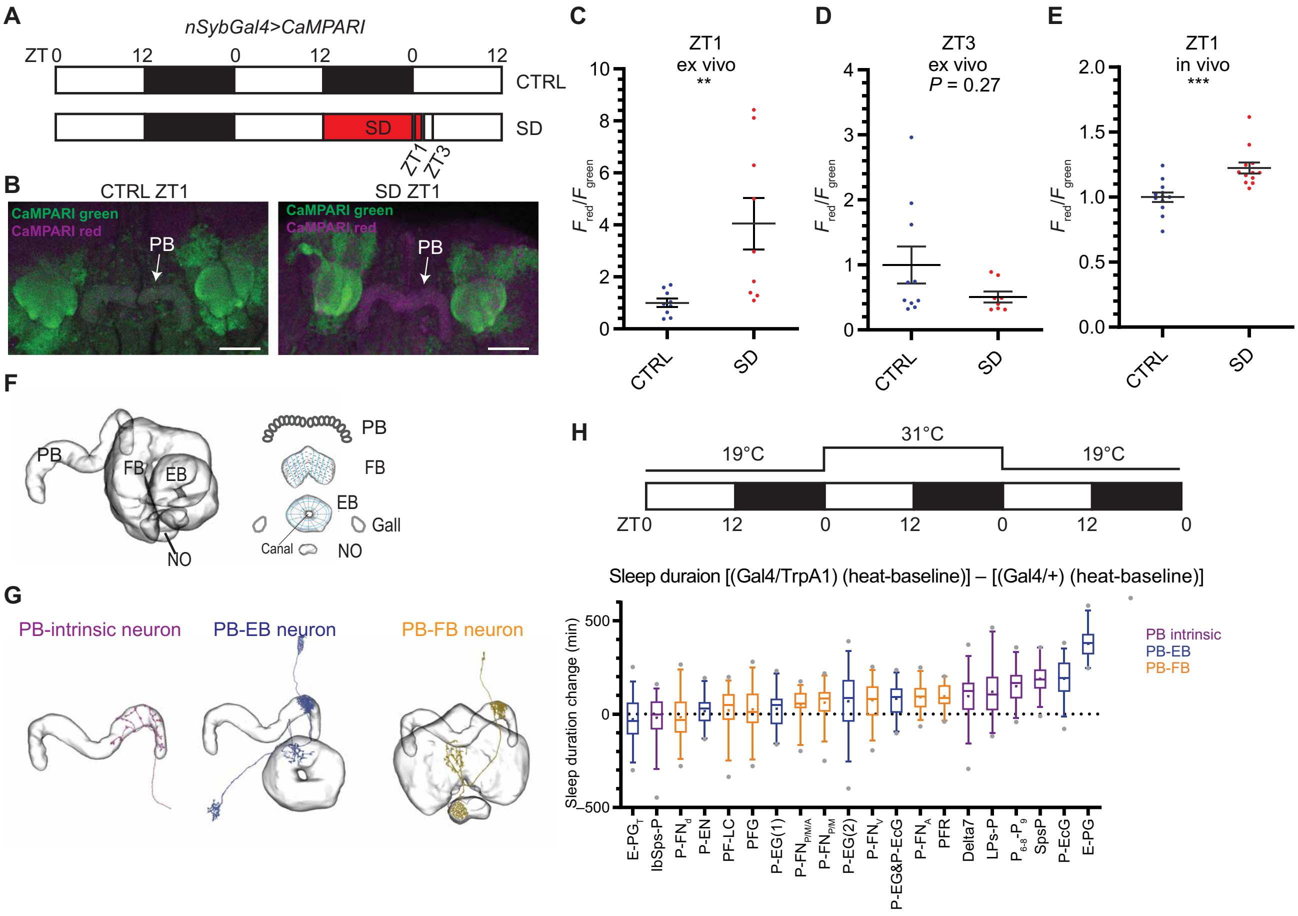

Fig. 1. PB intrinsic and PB-EB neurons are involved in sleep regulation. (**A**) A schematic representation of the sleep deprivation paradigm for CaMPARI imaging. Male nSybGal4>CaMPARI flies were sampled and imaged at ZT1 and ZT3. Open bar, light phase, ZT0 to ZT12; solid bar, dark phase, ZT12 to ZT0; red bar, sleep deprivation (SD). (**B**) Representative images of nSybGal4>CaMPARI imaging in the CTRL group (left) and the SD group (right) at ZT1. Green, CaMPARI green; magenta, CaMPARI red. Arrows, PB. Scale bars, 30 μm. (**C** to **E**) Quantification of the normalized F_{red}/F_{green} ratio of nSybGal4>CaMPARI in the PB in the CTRL (blue) and the SD (red) group by ex vivo [(C) and (D)] and in vivo (E) CaMPARI imaging at ZT1 [(C) and (E)] and ZT3 (D). Error bars, SEM. $^{**}P < 0.01$ and $^{***}P < 0.001$, Mann-Whitney test. (**F** and **G**) A schematic representation of the central complex components (F) and the representative projection patterns of three types of PB-projecting neurons (G). Illustration based on data from NeuPrint, licensed under CC BY 4.0 (https://creativecommons.org/licenses/by/4.0/). NO, noduli. (**H**) A schematic representation of the thermogenetic activation screen (top) and the quantification of the sleep duration changes (bottom). Fly sleep was monitored at 19°C during day 1, followed by thermogenetic activation at 31°C during day 2, and the flies returned to 19°C for recovery during day 3. Sleep duration changes were normalized to temperature change and Gal4 controls. Top three sleep-promoting drivers from right to left: E-PG (ss50574), P-EcG (ss02195), and SpsP (ss52267). Magenta, PB-intrinsic neurons labeling drivers; blue, PB-EB neurons labeling drivers; orange, PB-FB neurons labeling drivers. More than 30 male flies were measured in each group. Whiskers represent 5th to 95th percentiles.

with sleep drive in the PB, we carried out in vivo CaMPARI imaging, in which freely moving flies were exposed to UV to capture ongoing calcium activity. In this assay as well, sleep-deprived flies showed significantly higher PB calcium activity than the control group at ZT1 (Fig. 1E). The lower F_{red}/F_{green} ratio in the in vivo setting (Fig. 1E) relative to ex vivo (Fig. 1C) is likely due to the reduced penetration of UV light through the cuticle. Despite this difference, the consistency in the direction of the changes across both conditions supports that PB calcium levels correlate with sleep drive.

Thermogenetic activation screen of PB-expressing split drivers

The PB is a neuropil located near the posterior surface of the fly brain and is part of the central complex, which plays important roles in navigation, sensorimotor signaling, and sleep (*21*, *22*). The central complex is a set of highly interconnected neuropils in the center of the fly brain, consisting of the PB, FB, EB, the gall, and the noduli. The neuropils are highly structured: The PB consists of 18 glomeruli, 9 in each hemibrain; the FB consists of approximately eight layers and nine columns; the EB consists of 16 wedges (Fig. 1F). These three major components are connected by tangential neurons and columnar neurons: PB tangential neurons arborize across glomeruli; EB ring neurons arborize across wedges, whereas FB tangential neurons arborize across columns and projects to different layers. Columnar neurons arborize to one PB glomeruli, one EB wedge, or one FB column. Some neurons have additional projections to the gall or the noduli (*23*–*25*).

As mentioned above, other components of the central complex, most notably the FB (*26*) and the EB (*20*, *27*, *28*), also play important roles in sleep regulation. The sleep homeostasis process indicates that sleep drive increases with sleep deprivation and then leads to rebound sleep (*29*). If increased PB calcium is due to sleep deprivation and reflects enhanced sleep drive, then these neurons might promote sleep. However, which are the relevant neurons?

There are about 3000 neurons in the central complex, of which about 660 neurons arborize in the PB (*30*). They also connect to other central complex neurons to form highly ordered neural networks. To understand which subsets of PB-expressing neurons function in sleep regulation and to avoid targeting nonspecific neurons, we made use of the split-Gal4 library generated by Wolff and Rubin (*24*). These lines have greatly improved specificity than the traditional driver lines because Gal4 is only active in the intersection of the p65AD and Gal4DBD patterns (*31*). On the basis of their projection pattern/morphology, there are three categories of the PB-expressing neurons: (i) PB intrinsic neurons with most of their projections in the PB and no arborization in the EB or the FB; (ii) PB-EB neurons, which innervate the PB and the EB; and (iii) PB-FB neurons, which innervate the PB and the FB (Fig. 1G).

To identify which sets of PB-expressing neurons function in sleep regulation, we carried out a thermogenetic activation screen with the PB-expressing split-Gal4 library (Fig. 1H); each driver labeled a sparse subset of PB-expressing neurons. When comparing the sleep change upon thermo-activation between activated Gal4>dTrpA1 flies and Gal4/+ control flies, no wake-promoting driver was found in our thermogenetic activation screen of PB-expressing drivers (Fig. 1H). This is consistent with the increased CaMPARI signal in the PB upon sleep deprivation. Sleep was significantly increased when several groups of PB-EB connecting neurons and PB intrinsic neurons were thermogenetically activated. In contrast, activation of PB-FB connecting neurons had only a minor or negligible effect (Fig. 1H), suggesting that the PB and its connection with the EB have a more important role in sleep regulation than the connection between the PB and the FB. The top three drivers with the most robust sleep-increasing phenotypes are ss52267, ss50574, and ss02195. On the basis of the brain regions that they innervate, the relevant neurons labeled by these three drivers have been named by Wolff and Rubin (*24*) as the SpsP neurons [innervating the superior posterior slopes (SPSs) and the PB], the EPG neurons (innervating the EB, the PB, and the gall), and the PEcG neurons (innervating the PB, the EB canal, and the gall), respectively.

Four SpsP neurons promote sleep

As the SpsP driver (ss52267) only labels four SpsP neurons in the central complex and weakly labels a few additional cells in the brain and the ventral nerve cord (VNC) (Fig. 2A and fig. S2A), it is an attractive candidate for follow-up studies. Thermogenetic activation of the SpsP driver resulted in a daytime as well as a nighttime sleep increase (Fig. 2, B and C). Notably, activation did not alter locomotor activity during movement compared to the TrpA1/+ control group (Fig. 2D), suggesting that the sleep increase is not due to defective locomotion. Thermogenetic activation of SpsP also led to more consolidated sleep; sleep bout duration increased (Fig. 2E), and sleep bout number decreased (Fig. 2F). In addition, optogenetic activation of the SpsP driver caused a nighttime sleep increase (fig. S3, A and B). Blocking vesicle endocytosis and, thus, synaptic transmission in the SpsP neurons by expressing shibire^{ts} (*32*) also caused sleep loss at night from ZT14 to ZT21 (Fig. 2, G and H).

On the basis of the calcium increase in the PB with overnight sleep deprivation (Fig. 1C), we also assayed the SpsP neurons with CaMPARI imaging in flies after overnight sleep deprivation: Calcium in the SpsP cell bodies increased after overnight sleep deprivation (Fig. 2, I and J). Together, all of these results indicate that the SpsP neurons are important for sleep and that their activity increases with sleep drive.

SpsP neurons receive signals from and send signals to the PB

To explore the neural circuit basis of sleep regulation by SpsP neurons, we first took advantage of the recently published neuPrint analysis tool of fly brain electron microscopy (EM) reconstruction (*33*, *34*). The EM reconstruction was carried out in a large portion of the central brain of the fly (or referred to as "hemibrain"). The four SpsP neurons are all annotated in neuPrint (IDs: 881212361, 911893174, 941136727, and 941469110), and the EM reconstruction shows that each SpsP neuron expands its projections across all nine PB glomeruli of each half hemibrain. Moreover, there are two SpsP neurons that project to the PB in the left side of the hemibrain, and two SpsP neurons that project to the PB in the right side of the hemibrain (Fig. 3A). However, the EM reconstruction is missing the SpsP arborizations within the SPS region, probably because it was not carried out on a full brain. Synaptic connectivity analysis based on the EM reconstruction only identifies sparse SpsP connections, most of which remain within the central complex: SpsP neurons receive inputs from upstream neurons in the PB, including Delta7 and SpsP itself, and they send outputs to downstream neurons in the PB, including PB intrinsic neurons such as Delta7 and SpsP, PB-FB neurons such as P-FNd, and PB-EB neurons such as PEN (Fig. 3B).

We then labeled the dendrites of SpsP neurons with DenMark (*35*) and the axons with Syt.eGFP (*36*). Similar to the results from the EM reconstruction, the immunostaining showed that SpsP

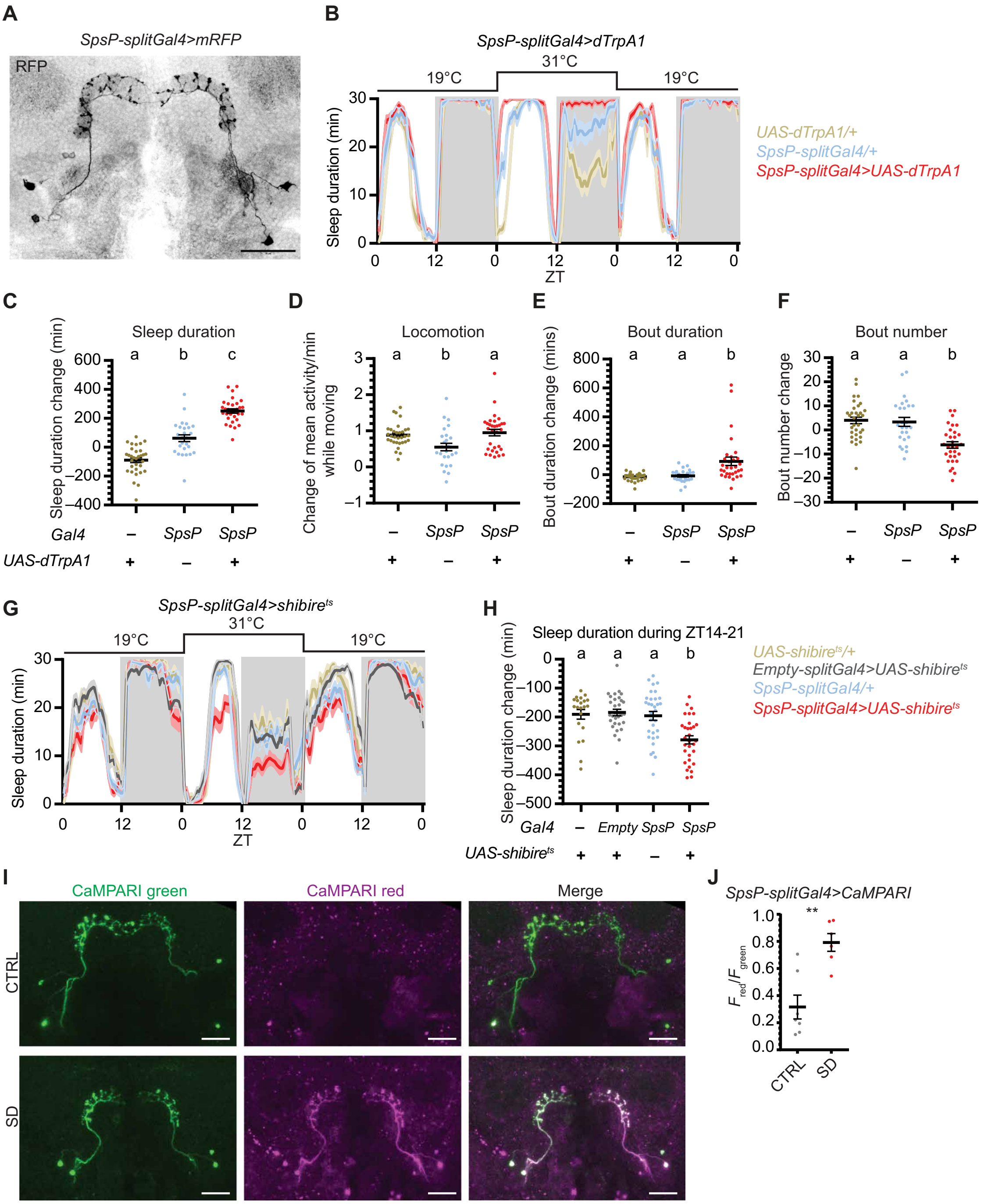

Fig. 2. Four SpsP neurons promote sleep. (**A**) Representative image of the four SpsP cells labeled by SpsP-splitGal4>mRFP stained for RFP. Scale bar, 30 μm. (**B** to **F**) Sleep profiles (B) and quantification of the changes in sleep duration (C), locomotion (D), sleep bout duration (E), and sleep bout number (F) from ZT0 to ZT24 upon thermogenetic activation of the SpsP neurons. Sleep profiles are averaged in 30-min bins. Changes were calculated by subtracting the value of baseline on day 1 from that of activation on day 2. Shaded area/error bars, SEM. Red, SpsP-splitGal4>UAS-dTrpA1 ($n = 31$); yellow, UAS-dTrpA1/+ ($n = 32$); blue, SpsP-splitGal4/+ ($n = 26$). Letters represent statistically distinct groups; $P < 0.01$, Kruskal-Wallis test followed by a post hoc Dunn's test. (**G** and **H**) Sleep profiles (G) and quantification of the changes in sleep duration during ZT14 to ZT21 (H) upon thermogenetic inhibition of the SpsP neurons. Sleep profiles are averaged in 30-min bins. Changes were calculated by subtracting the value of baseline from ZT14 to ZT21 on day 1 from that of inhibition from ZT14 to ZT21 on day 2. Shaded area/error bars, SEM. Red, SpsP-splitGal4>UAS-shibire^{ts} ($n = 31$); gray, Empty-splitGal4>UAS-shibire^{ts} ($n = 31$); yellow, UAS- shibire^{ts} /+ ($n = 21$); blue, SpsP-splitGal4/+ ($n = 32$). Letters represent statistically distinct groups; $P < 0.001$, Kruskal-Wallis test followed by a post hoc Dunn's test. (**I**) Representative images of SpsP-splitGal4>CaMPARI flies in the CTRL (top) group and the sleep deprivation (SD) (bottom) group of flies. Green, CaMPARI green; magenta, CaMPARI red. Scale bars, 30 μm. (**J**) Quantification of the CaMPARI F_{red}/F_{green} ratio in the cell bodies of the SpsP cells of SpsP-splitGal4>CaMPARI flies in the CTRL group ($n = 6$) and the SD group ($n = 7$). The average F_{red}/F_{green} was calculated for each fly. Each dot represents an individual fly. Error bars, SEM. $^{**}P < 0.01$, Mann-Whitney test. Male flies were used.

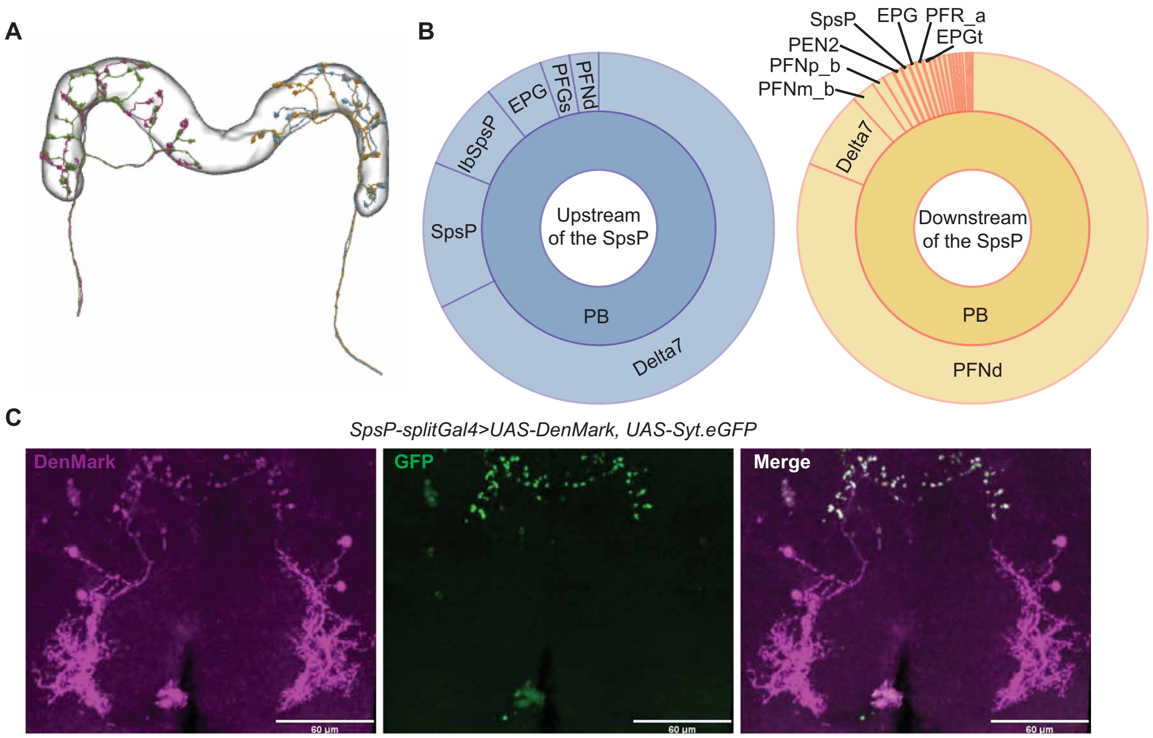

Fig. 3. SpsP neurons receive signals from and send signals to the PB. (**A**) The projection patterns of the four SpsP neurons in the PB based on the electron microscopy (EM) reconstruction from NeuPrint, licensed under CC BY 4.0 (https://creativecommons.org/licenses/by/4.0/). Each color represents an individual SpsP neuron. (**B**) Connectivity analysis of a representative SpsP neuron based on the EM reconstruction, adapted from NeuPrint. Left: Upstream (left) and downstream (right) brain regions and neurons of the representative SpsP neuron are shown. The size of each section represents the relative abundance of synaptic connections between the SpsP neuron and the specific type of neurons. (**C**) Representative images of the dendrites (left) and the axons (middle) of the SpsP cells labeled by SpsP-splitGal4>UAS-DenMark, UAS-Syt.eGFP stained for dsRed and GFP. Scale bars, 60 μm.

neuron dendrites receive signals in the SPS and PB, and SpsP neuron axons send signals to the PB (Fig. 3C).

SpsP neurons receive convergent activating signals from the EB via EPG neurons

Because both the EM reconstruction and immunostaining showed that most of the input and output signals of SpsP neurons lie within the PB, we postulated that most of the upstream and downstream neurons of SpsP neurons relevant to sleep regulation would be covered by our thermogenetic activation screen of PB-expressing split-Gal4 drivers. These neurons might include the EPG neurons, which caused the most robust sleep increase phenotype in the screen (Fig. 1H) and was indicated to lie upstream of the SpsP neurons by the connectivity analysis of the EM reconstruction (Fig. 3B).

Expression of mCD8-GFP (*37*) in the EPG driver labels about 20 pairs of EPG neurons in the brain (Fig. 4A), with only two additional neurons weakly labeled in the VNC (fig. S2B). Whereas the EPG driver labels all the glomeruli of the PB and the EB, EPG neurons are columnar neurons that tile together, so each individual EPG neuron is a columnar neuron that innervates one section of the EB, one of the 18 glomeruli of PB, and the gall (Fig. 4C). Immunostaining with DenMark and Syt.eGFP shows that the EPG dendrites are mostly in the EB and the axons in the PB (Fig. 4B), indicating that EPG neurons send signals from the EB to the PB. Consistent with this notion, connectomic analysis based on the EM reconstruction showed that EPG neurons lie upstream of SpsP neurons and form synapses with them. Thus, signals from different EPG neurons potentially converge onto each SpsP neuron via the PB neuropil.

Thermogenetic activation of the EPG driver (ss50574) resulted in a marked increase in daytime and nighttime sleep duration (Fig. 4, D and E) without significantly affecting locomotor activity (Fig. 4F). Similar to thermogenetic activation of the SpsP neurons, thermogenetic activation of the EPG neurons also resulted in less fragmented sleep, with the sleep bout duration significantly increased (Fig. 4G) and sleep bout number decreased (Fig. 4H). Optogenetic activation of the EPG neurons with CsChrimson expression similarly caused a consistent and robust sleep increase (fig. S4, A and B), indicating that the EPG neurons also promote sleep. However, blocking vesicle endocytosis with shibire^{ts} in EPG neurons had a puzzling effect by leading to a mild nighttime sleep increase rather than a decrease (fig. S4, C and D).

To further investigate the connection between the SpsP neurons and the EPG neurons, different components needed to be expressed in the two sets of neurons. To this end, we generated split-LexA lines to label these neurons; we verified that the new split-LexA lines had the identical expression patterns of the split-Gal4 lines by expressing LexAop-GFP and UAS-RFP simultaneously (fig. S5).

Using the split-LexA line, GFP was expressed in the EPG neurons and RFP expressed in the SpsP neurons simultaneously. This revealed many adjacent projections of the EPG neurons and the SpsP neurons in the PB (Fig. 4I), indicating a likely synaptic connection between them.

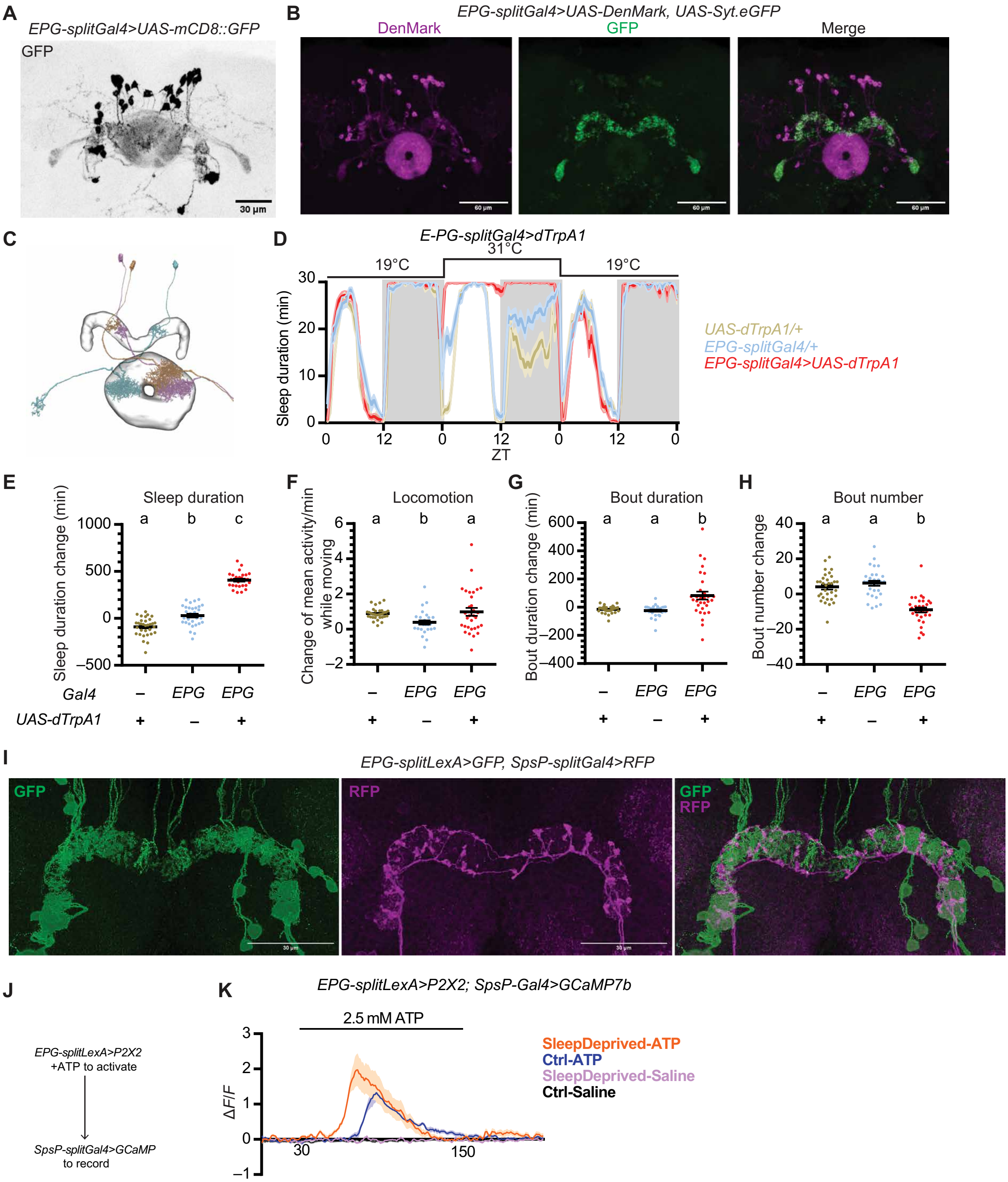

Fig. 4. SpsP neurons are downstream of EPG neurons. (**A**) Representative image of the EPG neurons labeled by EPG-splitGal4>UAS-mCD8::GFP stained for GFP. Scale bar, 30 μm. (**B**) Representative images of the dendrites (magenta) and the axons (green) of the EPG neurons labeled by EPG-splitGal4>UAS-DenMark, UAS-Syt.eGFP stained for dsRed and GFP. Scale bars, 60 μm. (**C**) Projection patterns of three representative EPG neurons based on the EM reconstruction from NeuPrint. Each color represents an individual EPG neuron. (**D** to **H**) Sleep profiles (D) and quantification of the changes in sleep duration (E), locomotion (F), sleep bout duration (G), and sleep bout number (H) from ZT0 to ZT24 upon thermogenetic activation of EPG neurons. Sleep profiles are averaged in 30-min bins. Changes were calculated by subtracting the value of baseline on day 1 from that of activation on day 2. Shaded area/error bars, SEM. Red, EPG-splitGal4>UAS-dTrpA1; yellow, UAS-dTrpA1/+; blue, EPG-splitGal4/+. Letters represent statistically distinct groups; $P < 0.0001$, Kruskal-Wallis test followed by a post hoc Dunn's test. More than 30 male flies were used for each group. (**I**) Representative images of EPG-splitLexA>LexAop-mCD8::GFP, SpsP-splitGal4>UAS-IVS-mCD8::RFP stained for dsRed and GFP. Scale bars, 30 μm. (**J**) Schematic representation of the experimental design to test functional connectivity from the EPG neurons to the SpsP neurons. (**K**) Average GCaMP traces ($\Delta F/F$) of SpsP cells in response to EPG activation. More than five male flies were used for each group. Shaded area, SEM.

We then assayed for a functional connection between the EPG neurons and the SpsP neurons by monitoring the levels of calcium and the second messenger cyclic adenosine 3′,5′-monophosphate (cAMP) in the SpsP neurons upon activation of EPG neurons. To monitor cAMP levels, we generated a 20xUAS-EPACH187 line, which is more sensitive to cAMP than the original EPAC sensor (*38*–*41*). GCaMP or EPACH187 was expressed in the SpsP neurons to record calcium or cAMP levels, and the adenosine 5′-triphosphate (ATP)–gated cation channel P2X2 was expressed in the EPG neurons (Fig. 4J).

Activation of EPG neurons with ATP perfusion caused a significant increase in both the calcium level and the inverse fluorescence resonance energy transfer (FRET) signal in the SpsP neurons (Fig. 4K and fig. S4E), indicating a functional connection from the EPG neurons to the SpsP neurons. Moreover, the calcium response is even stronger in flies that have been sleep deprived for 13 hours overnight compared to the control group (Fig. 4K), suggesting that the connection is further strengthened by sleep deprivation.

The connections from SpsP neurons to the PEcG neurons are dynamically modulated by sleep drive

Because the axons of the SpsP neurons are in the PB, we next asked which subset of PB-expressing neurons act downstream of the SpsP neurons to regulate sleep. Synaptic connectivity analysis based on the EM reconstruction indicated that one of the major cell types that lies downstream of the SpsP neurons is the P-FNd neurons (neurons that projects to the PB, the fan-shaped body, and the noduli), and SpsP neurons have been reported to act upstream of the P-FNd neurons in velocity tuning (*42*). However, the sleep profile of P-FNd activation is comparable to the control groups (fig. S6), suggesting that the SpsP neurons regulate sleep through a different circuit.

In our thermogenetic activation screen of PB-expressing split-Gal4 drivers, the PEcG driver (ss02195) showed the most robust sleep change among drivers that label neurons that receive signal inputs from the PB (Fig. 1H).

Expression of mCD8-GFP with the PEcG driver confirmed that it labels about nine pairs of PEcG neurons (Fig. 5A) in the brain with nothing detectable in the VNC (fig. S2C). PEcG neurons are canal P-EG neurons: A majority of their dendrites arborize in the PB, and their axons wrap the EB canal (Fig. 5B) (*24*), sending signals from the PB to the EB. Like the EPG neurons, each individual PEcG neuron is a columnar neuron that innervates one section of the EB, one glomerulus of the PB, and the gall (*24*). The PEcG neurons are not annotated by the EM reconstruction. Transsynaptic mapping of the SpsP neurons using trans-Tango (*43*) showed that the SpsP neurons send signals to neurons expressed in the EB and the PB (Fig. 5C), which is similar to the arborization pattern of the PEcG neurons. Moreover, the synaptic connections between the presynaptic sites of the SpsP neurons and the PEcG neurons were investigated by GFP Reconstitution Across Synaptic Partners (GRASP) (*44*) imaging. Presynaptic nSyb-spGFP1-10 was expressed in the SpsP neurons and CD4-spGFP11 expressed in the PEcG neurons simultaneously. Immunostaining of reconstituted GFP showed synaptic connections between the SpsP neurons and the PEcG neurons (Fig. 5D), supporting the notion that the SpsP neurons send signals to the PEcG neurons.

Like the SpsP neurons and the EPG neurons, thermogenetic activation of the PEcG neurons resulted in significant increases in both daytime and nighttime sleep duration (Fig. 5, E and F), with no significant difference in locomotor activity (Fig. 5G). Optogenetic activation of the PEcG neurons also caused a sleep increase (fig. S7), whereas blocking vesicle endocytosis in the PEcG neurons caused less nighttime sleep (Fig. 5, H and I), indicating that the PEcG neurons contribute to sleep regulation.

We then examined the functional connection between the SpsP neurons and the PEcG neurons. Activation of the SpsP neurons by ATP perfusion led to a significant cAMP increase (fig. S8) and calcium increase (Fig. 6, A and B) in the PEcG neurons. The calcium response is further increased in sleep-deprived flies (Fig. 6, A and B), indicating that this functional connection between the SpsP neurons and the PEcG neurons is tunable.

Are SpsP neurons altered upon sleep deprivation? Bruchpilot (BRP) is a key component of the presynaptic active zone, and the BRP abundance has been shown to correlate with vesicle release probability in *Drosophila* (*45*–*48*). Using Synaptic Tagging with Recombination (STaR) (*49*) to specifically tag the endogenous BRP protein with a V5 tag in the presynaptic sites of the SpsP neurons, we found that BRP is enriched in the SpsP neurons axons after sleep deprivation (Fig. 6, C and D). Together, these results indicate that the SpsP neurons act upstream of the PEcG neurons to promote sleep and that this connection is strengthened by sleep deprivation.

SpsP neurons integrate dopaminergic signaling to regulate sleep

A previous single-neuron labeling study with MultiColor FlpOut showed that individual SpsP neurons have abundant dendrites in the SPS (*23*). This suggests that these neurons might integrate other sleep-relevant signals in addition to conveying signals between the EB and the PB. Because dopaminergic signaling is an important wake-promoting molecule in *Drosophila* (*50*–*53*) and because several dopaminergic neuron clusters project to the SPS (*54*), we asked whether the SpsP neurons might also integrate dopaminergic signals to regulate sleep.

To this end, we first investigated whether the sleep-promoting SpsP neurons receive dopaminergic signaling. We made use of the DopR-Tango reporter (*55*) that fuses the Tango system (*56*) with UAS-DopR1 to report the sites of endogenous dopamine action. Expression of DopR-Tango driven by the SpsP driver confirmed that the SpsP cells receive dopaminergic signaling (Fig. 7A). Moreover, coexpression of GFP driven by SpsP-Gal4 and RFP driven by Dop2R-LexA shows that the two pairs of SpsP neurons colocalize with Dop2R-expressing neurons (Fig. 7B), whereas the other neurons labeled by the SpsP driver do not colocalize (fig. S9A). This indicates that that the SpsP neurons express the inhibitory dopaminergic receptor Dop2R. Consistent with the sleep-promoting phenotype of activating the SpsP cells, knocking down Dop2R with RNA interference (Fig. 7, C and D) or mutating the gene with CRISPR-Cas9 (fig. S9, B and C) in these cells (*57*) increased daytime sleep significantly. No increase in nighttime sleep was observed possibly because of the ceiling effect. This supports an inhibitory role of Dop2R in the SpsP cells. Rebound sleep was reduced after overnight sleep deprivation when Dop2R was down-regulated (Fig. 7, E and F, and fig. S9, D and E).

We interpret these results to indicate that dopamine signaling acts like a gate in the SpsP cells to regulate sleep. During regular daytime, the SpsP cells are inhibited by wake-promoting dopaminergic neurons through Dop2R to help keep flies awake, whereas this Dop2R effect is suppressed after sleep deprivation. This helps

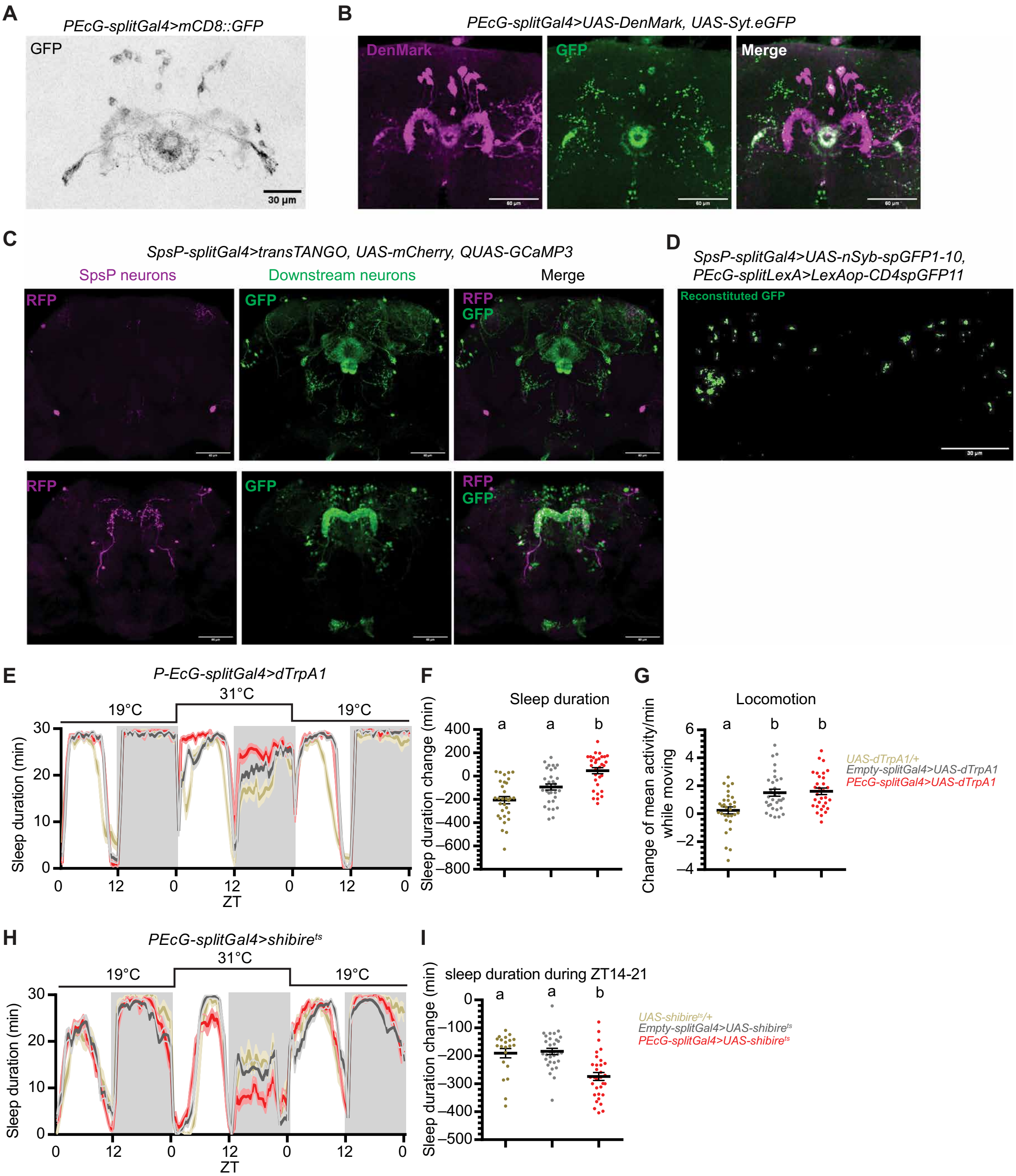

Fig. 5. SpsP neurons are upstream of sleep-promoting PEcG neurons. (**A**) Representative images of the PEcG neurons labeled by PEcG-splitGal4>UAS-mCD8::GFP stained for GFP. Scale bar, 30 μm. (**B**) Representative images of the dendrites (magenta) and the axons (green) of the PEcG neurons labeled by EPG-splitGal4>UAS-DenMark, UAS-Syt.eGFP stained for dsRed and GFP. Scale bars, 60 μm. (**C**) Representative images of SpsP neurons (magenta) and their downstream neurons (green) labeled by SpsP-splitGal4>transTANGO, UAS-mCherry; QUAS-GCaMP3 stained for dsRed and GFP. Scale bars, 60 μm. (**D**) Representative GRASP image of the synaptic connections in the PB between SpsP and PEcG neurons labeled by SpsP-splitGal4>UAS-nSyb-spGFP1-10, PEcG-splitLexA>LexAop-CD4spGFP11 stained for reconstituted GFP. Scale bar, 60 μm. (**E** to **G**) Sleep profiles (E) and quantification of the changes in sleep duration (F) and locomotion (G) from ZT0 to ZT24 upon thermogenetic activation of the PEcG neurons. Sleep profiles are averaged in 30-min bins. Changes were calculated by subtracting the value of baseline on day 1 from that of activation on day 2. Shaded area/error bars, SEM. Red, PEcG-splitGal4>UAS-dTrpA1; gray, Empty-splitGal4>UAS-dTrpA1; yellow, UAS-dTrpA1/+. Letters represent statistically distinct groups; $P < 0.01$, Kruskal-Wallis test followed by a post hoc Dunn's test. More than 30 male flies were used for each group. (**H** and **I**) Sleep profiles (H) and quantification of the changes in sleep duration during ZT14 to ZT21 (I) upon thermogenetic inhibition of the PEcG neurons. Sleep profiles are averaged in 30-min bins. Changes were calculated by subtracting the value of baseline from ZT14 to ZT21 on day 1 from that of inhibition from ZT14 to ZT21 on day 2. Shaded area/error bars, SEM. Red, PEcG-splitGal4>UAS-shibire^{ts} ($n = 32$); gray, Empty-splitGal4>UAS-shibire^{ts} ($n = 31$); yellow, UAS-shibire^{ts}/+ ($n = 21$). Letters represent statistically distinct groups; $P < 0.001$, Kruskal-Wallis test followed by a post hoc Dunn's test. Male flies were used.

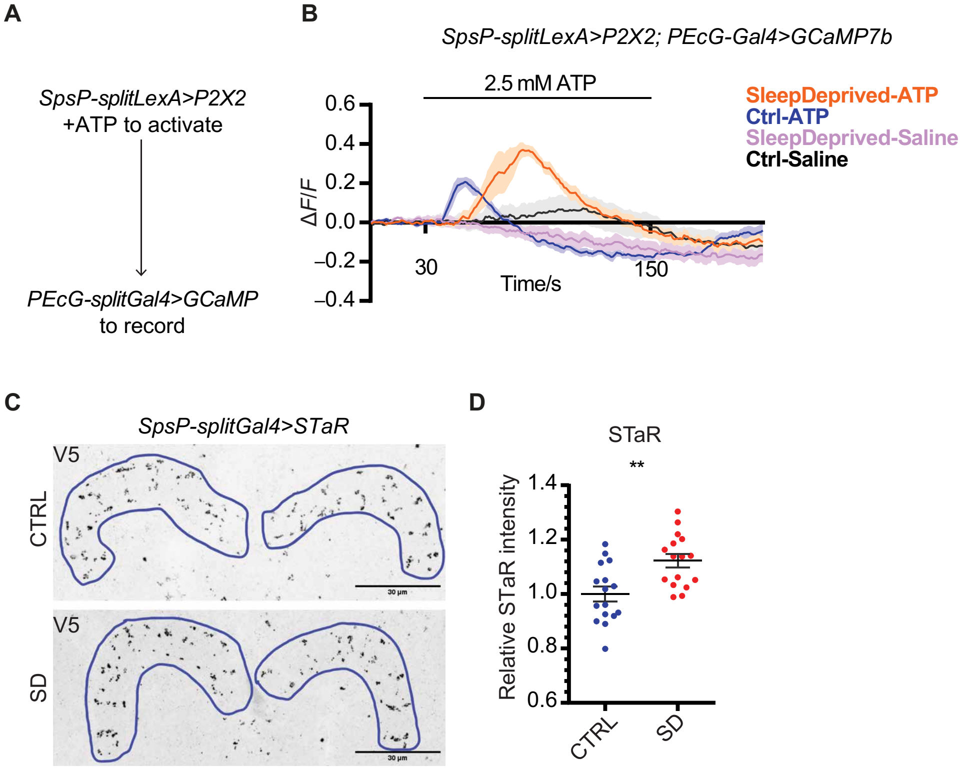

Fig. 6. Sleep deprivation changes connectivity strengths between SpsP neurons and PEcG neurons. (**A**) Schematic representation of the experimental design to test functional connectivity from the SpsP neurons to the PEcG neurons. (**B**) Average GCaMP traces (Δ*F*/*F*) of PEcG cells in response to SpsP activation in the control group of flies (Ctrl, *n* = 8) and flies after sleep deprivation (SleepDeprived, *n* = 8). Male flies were used. Shaded area, SEM. (**C** and **D**) Representative images (C) and quantification (D) of BRP protein abundance labeled by SpsP-splitGal4>UAS-Flp, brp(FRT.Stop)V5 stained for V5. Intensity was normalized to the average value of the control group. Examples of the regions of interest (ROIs) were drawn in blue. Eight male flies (16 hemi-PB ROIs) were used for each group. Scale bars, 30 μm. Error bars, SEM. Mann-Whitney test, ***P* < 0.01.

activate SpsP cells to drive rebound sleep. Dop2R down-regulating causes the SpsP cells to mimic a "rebound sleep" state and prevents them from mounting a greater sleep rebound response. Together, the results indicate that the SpsP neurons integrate dopaminergic signaling through Dop2R to further refine sleep regulation.

DISCUSSION

We have shown in this study that the activity of the PB increases with sleep deprivation and that the four key SpsP neurons that innervate this neuropil are sleep promoting. To carry out this function, these SpsP neurons also communicate with the EB and do so by sending signals through the PEcG neurons and by receiving signals from the EPG neurons (Fig. 8). Because the SpsP neurons also receive important wake-promoting inputs via dopamine signaling, we suggest that the SpsP neurons are a central player in fly brain sleep regulation.

An unbiased whole-brain calcium snapshot captures active brain regions in sleep-deprived flies

Through analysis of CaMPARI signal in multiple brain regions, the PB, along with the well-known sleep regulating brain regions the EB and the MB, showed a robustly increased calcium level in sleep-deprived flies. There was in contrast only a minimal effect in the vision processing optic lobe and the olfactory processing AL (fig. S1D). This revealed the PB as a central player in sleep regulation. The calcium activity increase in the PB with sleep drive was observed in both ex vivo and in vivo CaMPARI imaging (Fig. 1, C to E). Although the sleep drive-inducing mechanical stimuli used in our study have recently been shown to also induce sleep-independent effects (*58*), our findings are supported by other independent methods; they reduce the likelihood of sleep-independent artifacts. Nonetheless, it will be important in future studies to conduct CaMPARI imaging in sleep-deprived flies compared to that in yoked controls.

Whole-brain CaMPARI imaging has two major advantages relative to other calcium indicators and should be able to identify networks that regulate specific internal states or behaviors different from sleep drive. (i) It is irreversible, allowing the capture of calcium activity snapshots that compare groups of flies of different internal states without any external transient stimuli such as drug application or neuronal manipulation. (ii) It is noninvasive, enabling the in vivo imaging of freely moving flies, in contrast to the restricted mobilization used for traditional in vivo calcium imaging.

A sleep-promoting circuit between PB and EB inside the central complex

PB, EB, and FB are the three major components of the central complex. Several groups of central complex neurons, including dorsal FB neurons, helicon cells, and EB ring neurons, have been implicated in sleep regulation (*20*, *26*, *53*, *59*–*63*). However, some findings

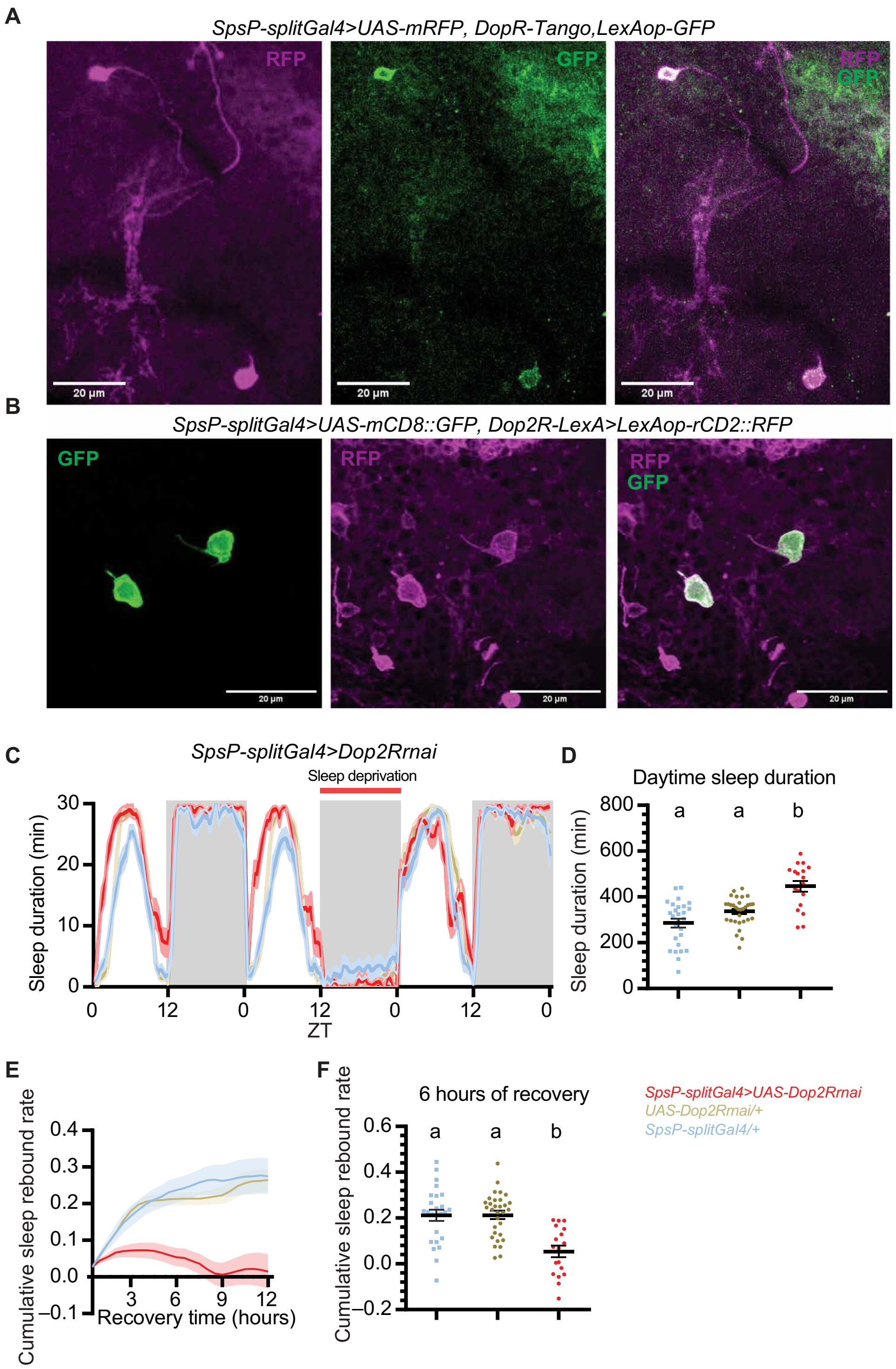

Fig. 7. SpsP neurons integrate dopaminergic signaling to regulate sleep. (**A**) Representative images of the SpsP neurons (magenta) and the site of dopaminergic signaling (green) labeled by SpsP-splitGal4>UAS-mRFP, DopR-Tango, LexAop-GFP stained for dsRed and GFP. Scale bars, 20 μm. (**B**) Representative images of the SpsP neurons (green) and the Dop2R-expressing neurons (magenta) labeled by SpsP-splitGal4>UAS-mCD8::GFP, Dop2R-LexA>LexAop-rCD2::RFP stained for dsRed and GFP. Scale bars, 20 μm. (**C** to **F**) Sleep profiles (C), quantification of the daytime sleep duration (D), cumulative sleep rebound rate curve in 12 hours (E), and the quantification of cumulative sleep rebound rate after 6 hours of recovery sleep (F) in the experimental group and the control groups of Dop2R knockdown in the SpsP neurons. [(C) and (E)] Average sleep duration in 30-min bins. Cumulative sleep rebound rate was calculated by first dividing the sleep rebound by the sleep loss for each fly and then cumulated over the bins. Shaded area/error bars, SEM. Red, SpsP-splitGal4>UAS-Dop2Rrnai ($n = 18$); yellow, UAS-Dop2Rrnai/+ ($n = 31$); blue, SpsP-splitGal4/+ ($n = 27$). Letters represent statistically distinct groups; $P < 0.01$, Kruskal-Wallis test followed by a post hoc Dunn's test. Virgin female flies were used.

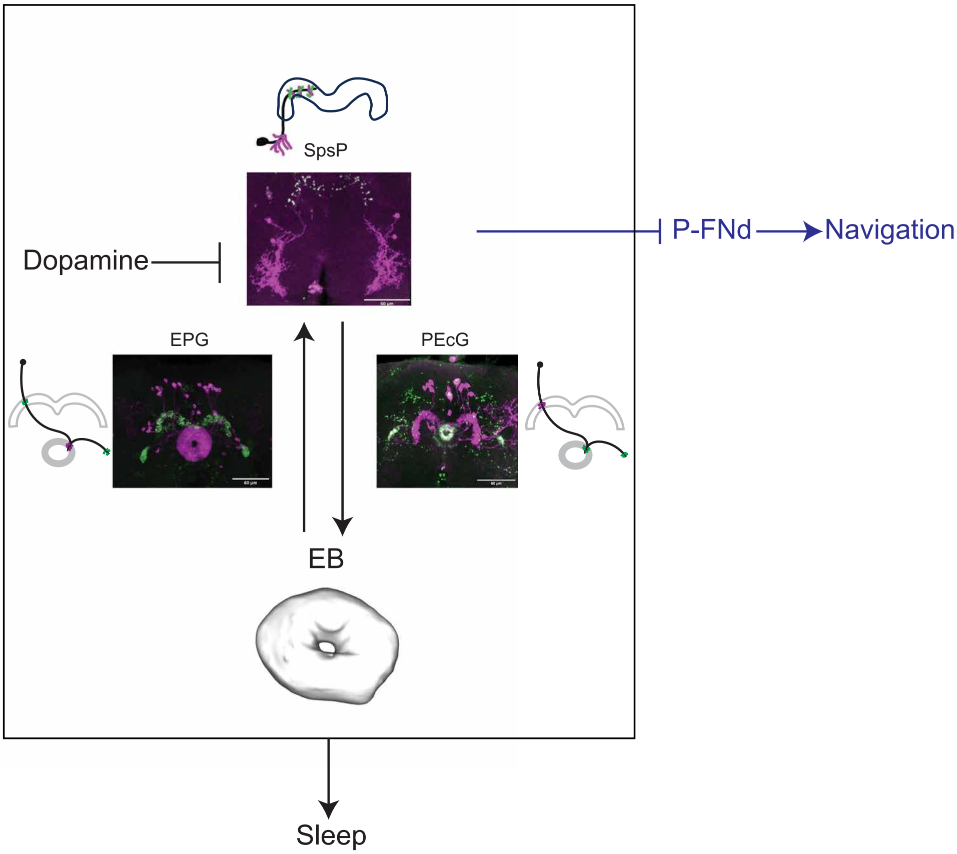

Fig. 8. Summary of the SpsP circuit in sleep regulation and navigation. Each SpsP neuron arborizes across PB glomeruli in the hemibrain; they integrate signals from the EB through multiple EPG neurons (each arborizes one PB glomerulus) and send signals to the EB through multiple PEcG neurons (each arborizes one PB glomerulus) to regulate sleep. In addition, SpsP neurons integrate inhibitory dopaminergic signaling probably from the SPS. The SpsP neurons also regulate navigation functions such as velocity tuning by inhibiting the P-FNd neurons (*42*, *78*). Confocal images are DenMark (magenta) and Syt.eGFP (green) expressed in the split driver. Cartoons represent the dendrites (magenta) and axons (green) of an individual neuron.

have recently been questioned due to the nonspecific targets of the Gal4 lines used (*64*–*66*), emphasizing the importance of using sparse-labeling Gal4 lines such as split-Gal4s. The role of PB neurons in sleep regulation has not been as extensively explored. Activation of PB-expressing drivers has only recently been shown to regulate sleep (*67*), but this study was limited by the broad expression patterns of the Gal4 drivers, within as well as outside the PB.

Here, we show that as few as four SpsP neurons are important for normal sleep regulation. This probably reflects their role in integrating signals from, and conveying signals to, larger groups of neurons. Recent characterization by light microscopy (*23*, *24*, *30*, *68*–*71*) and EM reconstruction (*25*) showed that the fly central complex is a highly organized structure. Each EPG and PEcG columnar neuron connects one EB section to a single PB glomerulus. In contrast, the tangential SpsP neurons span all nine pairs of PB glomeruli, acting as an integrating hub by gathering input from ~40 EPG neurons and sending output to ~18 PEcG neurons.

We present several lines of evidence supporting the relevance to sleep of these collecting and sending functions of the SpsP neurons. (i) Activating SpsP, EPG, or PEcG neurons significantly increased sleep (Figs. 2, B and C, 4, D and E, and Fig. 5, E and F). Although inhibition phenotypes were weaker, blocking synaptic transmission in SpsP or PEcG neurons significantly decreased sleep (Figs. 2G and 5H). Notably, previous studies have shown sleep increases induced by activating other central complex sleep regulators like the dFB or EB-R5 neurons, but, to our knowledge, inhibiting these neurons has not been reported to cause sleep effects. Unexpectedly, blocking synaptic transmission in the EPG neurons caused increased sleep (fig. S4, C and D), probably because they are functionally more heterogeneous than the less numerous SpsP and PEcG neurons. Consistent with this interpretation, the variation of locomotion and sleep bout duration is bigger in EPG-activated flies than the control groups (Fig. 4, F and G). (ii) The SpsP dendrites (Fig. 3C) and the EPG axons (Fig. 4B) are juxtaposed, as are the SpsP axons (Fig. 3C) and the PEcG dendrites (Fig. 5B). (iii) TransTango imaging showed that the downstream neuron targets of the SpsP neurons innervate the EB and PB (Fig. 5C). (iv) GRASP imaging confirmed synaptic connections between the SpsP neurons presynaptic sites and PEcG neurons within the PB (Fig. 5D). (v) EPG neuron activation increased SpsP calcium (Fig. 4K) and cAMP levels (fig. S4E), and SpsP activation increased PEcG calcium (Fig. 6B) and cAMP levels (fig. S8).

The circuit of EPG-SpsP-PEcG identified here suggests that information flow from PB-to-EB as well as its reverse, EB-to-PB, functions in sleep regulation. This substantially expands the understanding of how the central complex regulates sleep. The EB comprises ring neurons (EB-R1 to EB-R5) and columnar neurons (e.g., EPG and PEcG).

EB-R5 neurons (originally termed as R2 neurons) are known to encode sleep drive (*20*, *63*) and integrate circadian signals (*72*–*74*). These neurons act upstream of dFB–helicon cell–EB-R5 circuit (*20*, *59*), which forms a recurrent circuit for sleep homeostasis regulation.

This aligns with our SpsP-centric circuit: EB-R5 neurons likely regulate sleep via the EPG neurons (*20*, *28*, *63*), which convey signals to the SpsP neurons. The PEcG neurons, acting downstream of SpsP, project back to the EB, potentially influencing multiple sleep-regulating ring neuron groups.

SpsP neurons receive dopaminergic signaling to regulate sleep

In addition to communicating signals with the EB, the SpsP neurons respond to other inputs. DopR-Tango imaging shows that dopaminergic signaling affects these neurons (Fig. 7A). Dop2R-LexA labels the four SpsP neurons but not the other cells of the SpsP split Gal4 driver (Fig. 7B and fig. S9A). Moreover, Dop2R down-regulation within SpsP neurons increased daily sleep and decreased recovery sleep after sleep deprivation (Fig. 7, C to F, and fig. S9, B to E), indicating that SpsP neurons probably receive inhibitory signals from dopaminergic neurons to reduce sleep. Might the inhibitory dopaminergic signal come from the PB, perhaps from the one pair of T1/LPs-P dopaminergic neurons that innervates PB (*23*, *24*, *30*, *75*)? Probably not, as we found that both thermogenetic and optogenetic activation of the LPs-P neurons (labeled by ss52578) caused a sleep increase rather than a decrease, and optogenetic inhibition of the LPs-P neurons showed no sleep effect (fig. S9, F to H). This indicates that, if a connection exists between the LPs-P neurons and SpsP neurons, then it is excitatory, i.e., the opposite of what is indicated by the Dop2R KO phenotype. The SpsP neurons more likely receive dopaminergic input from within the SPS, where their arbors coincide with those from PAL, PPL2ab, and PPL2c dopaminergic neurons (*54*).

Integration of sleep and navigation

The four SpsP neurons share two uncommon features with the EPG neurons: (i) Their activity increases with sleep deprivation, and they are sleep-promoting; and (ii) they are also involved in navigation. EPG neurons are compass neurons that represent heading direction (*76*, *77*), and SpsP neurons function in velocity tuning (*42*). This function of SpsP neurons and that of sleep-promotion occur through distinct downstream circuits: the neurons send signals to the FB through the P-FNd neurons to regulate velocity tuning (*42*, *78*) and send signals to the EB through PEcG neurons to regulate sleep. How do the central complex and more specifically its four SpsP neurons coordinate these two very different functions? One possibility is that their plasticity is gated by sleep and wake states (*79*). Our data indicate that enhanced sleep drive caused multiple changes in the SpsP circuit: After sleep deprivation, the calcium activity of all four SpsP neurons increased (Fig. 2, I and J), BRP protein is more abundant in the SpsP axons (Fig. 6, C and D), and the functional connections of EPG-to-SpsP and SpsP-to-PEcG are stronger (Figs. 4, J and K, and 6, A and B). Similarly, connections of EBR5-to-EPG have been reported to increase with sleep deprivation (*28*). The calcium response of EPG-to-SpsP is also faster in sleep-deprived flies, whereas the calcium response of SpsP-to-PEcG is slower. Although calcium response kinetics are not typically used as a primary parameter in calcium imaging, this difference indicates that plasticity in the connection might occur not only in amplitude but also in response latency. The faster and slower changes might balance each other and make the total speed of EPG-SpsP-PEcG circuit consistent. The functions that these kinetic changes might serve are certainly of future interest.

We suggest that these changes integrate sleep and navigation in opposite directions: (i) They turn down navigation when an animal is about to sleep. The increase of activity and BRP abundance in the EBR5-EPG-SpsP circuit caused by sleep deprivation is accompanied by strong inhibition of the P-FNd neurons; this impairs velocity tuning and visual signal processing. (ii) Given the known role of the EPG neurons and SpsP neurons in navigation, the opposite gate might be enhanced by extensive navigation. For example, when the animal encounters a new environment, it might strengthen the connection between the SpsP and P-FNd neurons at the expense of the SpsP-PEcG connection and sleep. Perhaps the compact nature of the small fly brain necessitates such switches. Verifying these gates and then understanding the underlying switching mechanisms are fascinating topics for future study.

MATERIALS AND METHODS

Fly stocks and rearing

All flies were raised at 25°C on standard cornmeal food with a 12-hour:12-hour light:dark cycle. The genotypes of fly strains used are listed in Table 1.

Generation of fly lines

To generate the split-LexA lines, pBPZpGAL4DBDUw (Addgene, no. 26233) was firstly digested with Kpn I [New England Biolabs (NEB), no. R3142S] and Hind III (NEB, no. R0104S), and, then, Gal4DBDZp was replaced with LexADBDZp amplified from UAS-LexADBD (*80*) by Gibson assembly (NEB, no. E2611S) to generate a pBPLexADBDZp construct. The corresponding enhancer of each split driver was respectively amplified from the genomic DNA of wild-type flies and ligated into Aat II (NEB, no. R0117S) and Nae I (NEB, no. 0190S) digested pBPLexADBDZp construct. Sequencing-verified enhancer-LexADBDZp plasmids were injected into attP1 site on the second chromosome by Rainbow Transgenic Flies Inc. (Camarillo, CA, USA). Positive transformants were screened by eye color and confirmed by polymerase chain reaction (PCR). Enhancer-LexADBDZp flies were then recombined with corresponding p65ADZp lines that were inserted at attP40 site on the second chromosome to make a stable split-LexA line. Flies were confirmed by genotyping with the following primers: p65AD genotyping: DSCP-F (5′-GAGCTCGCCCGGGGATC-3′) + p65AD-R (5′-CGAGATCGGTGAATACGGCA-3′) with an expected band of 785 base pairs (bp); LexADBD genotyping: DSCP-F + LexADBD-R (5′-TTACAGCCAGTCGCCGTT-3′) with an expected band of 953 bp.

To generate the UAS-EPACH187 line, mCD8::GFP was cut out from pJFRC7-20XUAS-IVS-mCD8::GFP (Addgene, no. 26220) (*81*) by Not I and Xba I and replaced with EPAC-S-H187 from Epac-S-H187 (Addgene, no. 170348) (*38*). Sequencing-verified UAS-EPACH187 plasmids were injected into attP1 site on the second chromosome by Rainbow Transgenic Flies Inc. (Camarillo, CA, USA). Positive transformants were screened by eye color and confirmed by PCR.

Generated constructs and fly strains in this study are available upon request.

Sleep monitoring and analysis with DAM system

Flies of 5 to 10 days old were collected and loaded into glass behavioral tubes containing food of 5% sucrose and 2% agar. The tubes

Table 1. Resources of fly strains used in this study.

Figure	Fly strains	Source	Identifier	Note
Fig. 1	w*; UAS-CaMPARI (attP40); GMR57C10-Gal4 (VK00040), GMR57C10-Gal4 (VK00020)	Fosque *et al.* (*15*)	BDSC_58763	
	E-PG$_T$: w[1118]; R15E01-p65AD (attP40); VT016114-Gal4DBD (attP2)	Wolff and Rubin (*24*)	BDSC_75922	Janelia SS02254
	IbSps-P: w[1118]; R47G08-p65AD (attP40); VT012791-Gal4DBD (attP2)		BDSC_75846	Janelia SS04778
	P-FN$_d$: w[1118]; R16D01-p65AD (attP40); R15E01-Gal4DBD (attP2)		BDSC_75854	Janelia SS00078
	P-EN: w[1118]; VT026664-p65AD (attP40); VT032907-Gal4DBD (attP2)		BDSC_86624	Janelia SS54295
	PF-LC: w[1118]; VT000454-p65AD (attP40); VT001980-Gal4DBD (attP2)		BDSC_75926	Janelia SS02239
	PFG: w[1118]; VT017491-p65AD (attP40); VT000355-Gal4DBD (attP2)		BDSC_86628	Janelia SS52590
	P-EG(1) : w[1118]; VT000355-p65AD (attP40); VT040589-Gal4DBD (attP2)		BDSC_75924	Janelia SS02191
	P-FN$_{P/M/A}$: w[1118]; VT039497-p65AD (attP40); R83D12-Gal4DBD (attP2)		BDSC_86616	Janelia SS52245
	P-FN$_{P/M}$: w[1118]; VT039497-p65AD (attP40); R48A11-Gal4DBD (attP2)		BDSC_86596	Janelia SS52244
	P-EG(2) : w[1118]; R33A12-p65AD (attP40); VT040589-Gal4DBD (attP2)		BDSC_75811	Janelia SS27853
	P-FN$_V$: w[1118]; VT063307-p65AD (attP40); VT019742-Gal4DBD (attP2)		BDSC_86625	Janelia SS52577
	P-EG&P-EcG: w[1118]; VT000355-p65AD (attP40); R89F06-Gal4DBD (attP2)		BDSC_75928	Janelia SS02198
	P-FN$_A$: w[1118]; R16D01-p65AD (attP40); VT016114-Gal4DBD (attP2)		BDSC_75923	Janelia SS02255
	PFR: w[1118]; R38B06-p65AD (attP40); VT027015-Gal4DBD (attP2)		BDSC_86603	Janelia SS54549
	Delta7: w[1118]; VT019012-p65AD (attP40); R38G02-Gal4DBD (attP2)		BDSC_86723	Janelia SS52266
	LPs-P: w[1118]; VT019012-p65AD (attP40); R38G02-Gal4DBD (attP2)		BDSC_86626	Janelia SS52578
	P$_{6–8}$-P$_9$: w[1118]; R24A02-p65AD (attP40); R18G01-Gal4DBD (attP2)		BDSC_75856	Janelia SS00117
	SpsP: w[1118]; VT019012-p65AD (attP40); R72C10-Gal4DBD (attP2)		BDSC_86722	Janelia SS52267
	P-EcG: w[1118]; VT000355-p65AD (attP40); R47A08-Gal4DBD (attP2)		BDSC_75868	Janelia SS02195
	EPG: w[1118]; VT026664-p65AD (attP40); VT032907-Gal4DBD (attP2)		BDSC_86602	Janelia SS50574
	UAS-dTrpA1	Kang *et al.* (*87*)	N/A	
Fig. 2	UAS-mRFP	Bloomington Drosophila Stock Center	BDSC_7119	
	Empty-splitGal4: w[1118]; P65ad (attP40); Gal4DBD (attP2)	Hampel *et al.* (*88*)	BDSC_79603	
	20xUAS-shibire^{ts}-p10 (attp5)	Pfeiffer *et al.* (*89*)	N/A	
	UAS-CaMPARI2 (su(Hw)attP5); UAS-CAMPARI2 (VK00005)	Moeyaert *et al.* (*16*)	BDSC_78317; BDSC_78316*	
Fig. 3	UAS-DenMark, UAS-Syt.eGFP	Nicolai *et al.* (*35*)	BDSC_33065	
Fig. 4	UAS-mCD8::GFP	Lee and Luo (*37*)	N/A	
	UAS-mCD8::RFP (attP18), LexAop-mCD8::RFP (su(Hw)attP8)	Pfeiffer *et al.* (*81*)	BDSC_32229	
	EPG-splitLexA: VT026664-p65AD (attP40), VT032907-LexADBD (attP1)	This study	N/A	
	LexAop-P2X2	Yao *et al.* (*90*)	N/A	
	UAS-IVS-jGCaMP7b (VK00005)	Bloomington Drosophila Stock Center	BDSC_79029	
Fig. 5	transTANGO	Talay *et al.* (*43*)	BDSC_77123	
	QUAS-GCaMP3	Bloomington Drosophila Stock Center	BDSC_52231	
	UAS-nSyb-spGFP1-10, LexAop-CD4-spGFP11	Macpherson *et al.* (*44*)	BDSC_64314	
Fig. 6	SpsP-splitLexA: VT019012-p65AD (attP40), R72C10-LexADBD (attP1)	This study	N/A	
	UAS-STaR	Chen *et al.* (*49*)	BDSC_55751	
Fig. 7	UAS-mRFP, DopR-Tango, LexAop-GFP	Inagaki *et al.* (*55*)	N/A	
	LexAop2-rCD2::RFP, UAS-mCD8::GFP	Bloomington Drosophila Stock Center	BDSC_67093	
	Dop2R-LexA	Deng *et al.* (*91*)	N/A	
	UAS-Dop2Rrnai	Perkins *et al.* (*92*)	BDSC_50621	
Fig. S2	UAS-EGFP	Bloomington Drosophila Stock Center	BDSC_5430	
Fig. S3	UAS-CsChrimson-mVenus (attP18)	Bloomington Drosophila Stock Center	BDSC_55134	

(*Continued*)

(*Continued*)

Figure	Fly strains	Source	Identifier	Note
Fig. S4	UAS-EPACH187 (VK00027)	This study	N/A	
Fig. S5	PEcG-splitLexA: VT000355-p65AD (attP40); R47A08-LexADBD (attP1)	This study	N/A	
Fig. S9	UAS-Cas9.P2 (attP40)	Bloomington Drosophila Stock Center	BDSC_58985	
	UAS-Dop2RgRNA	Schlichting *et al.* (*57*)	N/A	
	UAS-GtAcr1-YFP (attP2)	Mohammad *et al.* (*93*)	BDSC_92983	

*A stable line with UAS-CaMPARI2 on the second and third chromosomes was made and crossed to the Gal4 line for Fig. 2 (I and J).

were then put onto the Drosophila Activity Monitors (DAM; TriKinetics Inc., Waltham, MA) system in an incubator of 25°C unless noted otherwise. Locomotion and sleep were analyzed by the 2020 version of Sleep and Circadian Analysis MATLAB Program (SCAMP) MATLAB Program.

Thermogenetic neuronal manipulation

Flies were collected, loaded into the DAM system, and entrained at 19°C with a 12-hour:12-hour light:dark cycle. Baseline sleep was measured at 19°C for at least 1 day and then transferred to 31°C for thermogenetic activation or inhibition. The temperature was then reduced back to 19°C for recovery measurements. Changes were calculated by subtracting the value of the baseline from the manipulation and then compared between groups.

For the thermogenetic activation mini-screen of PB-expressing neurons, the sleep duration change was calculated and plotted by first subtracting the sleep durations on day 2 (activation) by the sleep durations on day 1 (baseline) for each fly, and, then, the average sleep duration change of the Gal4/+ control group was subtracted from each fly in the corresponding Gal4/dTrpA1 group.

Sleep deprivation

Flies were collected and loaded into the DAM system, and, then, the DAM boards were loaded onto a vortexer mounting plate (TriKinetics Inc., Waltham, MA) mounted to a VWR shaker. Flies were vortexed for 2 s every ~20 s (uniformly distributed randomized) during the sleep deprivation period (*82*). Cumulative rebound rate of each individual fly was calculated as cumulative rebound rate = $\sum_{i=1}^{n} \frac{\text{SleepPostSD}(i) - \text{BaselineSleep}(i)}{\text{TotalBaselineSleep} - \text{TotalSleepDuringSD}}$, where i is the bin being analyzed, and sleep was analyzed in 30-min bins, with 48 bins in each day. TotalBaselineSleep was calculated as the total sleep duration of the corresponding ZT time to sleep duration on the baseline day. For example, when sleep is deprived from ZT12 to ZT0, sleep duration from ZT12 to ZT0 on the day before was counted as TotalBaselineSleep.

Optogenetic neuronal manipulation

Optogenetic experiments were carried out in the video-recording FlyBox (*83*). Fly food consisting of 5% sucrose, 2% agar, and 400 μM all trans-retinal (ATR) was dispensed into 96-well plates. ATR (40 mM) stock was made by dissolving the ATR powder (Sigma-Aldrich) in ethanol and stored in the dark at −20°C. Flies were transferred to the 96-well plates 4 days before the optogenetic stimulation. Each fly was put in an individual well. Flies were entrained with 12-hour:12-hour light:dark at 5 lux. Red light (LEDSupply, 624 to 634 nm, 0.1 mW mm^{-2}) pulsing at 5 Hz was used for CsChrimson experiments. Green light (LEDSupply, 520 to 540 nm, 0.1 mW mm^{-2}) was used for GtAcr1 experiments. The activity of the flies was captured every 10 s by WebCamImageSave (NirSoft). Locomotion was then analyzed by the PySolo software (*84*), trimmed into DAM readable format, and sleep was analyzed by the 2020 version of SCAMP (*85*).

Immunohistochemistry and confocal imaging

Flies at 5 to 10 days old were dissected in 1× PBS, and the brains and VNC were then fixed in 4% paraformaldehyde in PBS for 55 min. Tissues were then washed three times for 10 min in 0.5% Triton X-100 in PBS (PBT), blocked in PBT with 10% normal goat serum (NGS) at 4°C overnight, and incubated with primary antibodies in PBT with 10% NGS at 4°C overnight. Tissues were then washed three times for 15 min in PBT and incubated with corresponding secondary antibodies in PBT with 10% NGS at 4°C overnight. Next, tissues were washed three times for 15 min in the PBT and 15 min in the PBS, mounted in VectaShield mounting medium, and imaged using Leica Stellaris 8 confocal microscope with a white light laser. Images were processed and analyzed with ImageJ.

For GFP and RFP immunostaining, chicken anti-GFP (Abcam, catalog no. ab13970, RRID: AB_300798; 1:1000) and rabbit anti-dsRed (Takara Bio, catalog no. 632393, RRID: AB_3083500; 1:300) were used. For GRASP experiments, the reconstituted GFP was immunostained with rabbit anti-recombinant GFP (Thermo Fisher Scientific, no. G10362, RRID:AB_2536526; 1:100) and Alexa Fluor 488 anti-rabbit (Thermo Fisher Scientific, catalog no. A-11008, RRID:AB_143165; 1:400). For STaR experiments, the V5 tag was immunostained with mouse anti V5-tag conjugated to DyLight 550 (Bio-Rad catalog no. MCA1360D550GA, RRID:AB_2687576; 1:400).

CaMPARI imaging

For ex vivo CaMPARI imaging, brains were first dissected in adult hemolymph-like saline (AHL), and UV light (LEDSupply, 400 to 410 nm) was then illuminated onto freshly dissected brains rinsed in AHL (fig. S1A, bottom). The brains were then imaged using Leica Stellaris 8 confocal microscope. The UV light was pulsing in a 500-ms on and 200-ms off cycle (fig. S1C, top), this cycle was on for 60 s and then off for 30 s with a duration of 150 s in total to avoid

overheating (fig. S1C, bottom) for a total duration of 150 s. The paradigm was verified by high K^+ buffer application or dTrpA1 expression and activation.

For in vivo CaMPARI imaging, flies were put into a Sylgard plate covered by a piece of qPCR film, holes were poked in the film to allow the flies to breathe (fig. S1A, top). The UV light was pulsing in the same pattern as ex vivo CaMPARI imaging but with a duration of 900 s. Flies were then dissected, brains were fixated in 4% paraformaldehyde for 2 hours, washed three times with wash buffer for 15 min each time, mounted onto a glass slide, and imaged.

To compare the fluorescence signals between samples of different conditions, same settings of laser and detectors were used during each experiment. Sleep of each fly was first analyzed and picked before imaging, flies that had normal sleep were picked for the CTRL group, and flies that were sleep deprived were picked for the SD group. The signals were quantified by ImageJ, and F_{red}/F_{green} was calculated using the method in Edward *et al.* (*17*). Briefly, background was subtracted from each channel and then the F_{red}/F_{green} ratio was calculated.

GCaMP imaging

Male flies of 5 to 10 days old were used. Brains were dissected in ice-cold AHL and then mounted onto small pieces of poly-L-lysine–coated coverslips (Neuvitro, no. GG-12-PLL) on SYLGARD 184–coated Siskiyou perfusion chambers (Automate Scientific). ValveBank II perfusion system (Automate Scientific) was used to set up the perfusion flow. Briefly, solutions of AHL, ATP, and 85 mM K^+ buffer were put in individual channels. The flow rate was set to 1 drop per second. The brain was bathed in AHL before the focal plane was set. Each brain was put through three sets of flow consequentially: (i) AHL-AHL-AHL for a saline control, switching between two channels of AHL; (ii) AHL-2.5 mM ATP-AHL, switching between a channel of AHL and a channel of 2.5 mM ATP solution flow for stimulation, followed by AHL flow for recovery and wash; and (iii) AHL-85 mM K^+ buffer as a positive control. Only brains that were responding to 85 mM K^+ buffer were selected for analysis of the saline control and the response to 2.5 mM ATP stimulation.

UAS-GCaMP7b and UAS-mRFP were recombined and imaged at the same time, mRFP serving as an endogenous control. Images were captured one frame per second on Leica Stellaris 8 confocal microscope, analyzed with custom MATLAB program modified from Adel *et al.* (*86*). The change of mRFP over time was subtracted from GCaMP for correction. $\Delta F/F0$ of the GCaMP signal was then calculated as $(F - F0)/F0$, where F is the signal at the given frame, and F0 is the baseline signal that is defined as the average signal of the first 10 frames.

cAMP imaging

Male flies of 5 to 10 days old were used for cAMP imaging using the EPAC FRET sensor. The perfusion protocol was similar with that of GCaMP imaging, but 20 μM forskolin (cAMP agonist) was used as positive control instead of 85 mM K^+ buffer. Only brains that were responding to 20 μM forskolin were selected for analysis of the saline control and the response to 2.5 mM ATP stimulation.

For analysis, background-subtracted cyan fluorescent protein (CFP) and yellow fluorescent protein (YFP) signals were measured for every frame, and the inverse FRET (iFRET) signals were calculated with methods modified from Shafer *et al.* (*39*) to reflect the cAMP level: $F = \text{CFP}/[\text{YFP} - (\text{CFP} \times 0.27)]$, where 0.27 is the measured CFP spillover into the YFP channel in our scope settings. $\Delta F/F$ of the iFRET signal was then calculated as $(F - F0)/F0$, where F is the signal at the given frame and $F0$ is the baseline signal that is defined as the average signal of the first 10 frames.

Statistical analysis

Statistical tests were performed using the Statistics Kingdom website.

Supplementary Materials

This PDF file includes:

Figs. S1 to S9

REFERENCES AND NOTES

1. M. R. Zielinski, J. T. McKenna, R. W. McCarley, Functions and mechanisms of sleep. *AIMS Neurosci.* **3**, 67–104 (2016).
2. J. C. Hendricks, S. M. Finn, K. A. Panckeri, J. Chavkin, J. A. Williams, A. Sehgal, A. I. Pack, Rest in *Drosophila* is a sleep-like state. *Neuron* **25**, 129–138 (2000).
3. P. J. Shaw, C. Cirelli, R. J. Greenspan, G. Tononi, Correlates of sleep and waking in *Drosophila* melanogaster. *Science* **287**, 1834–1837 (2000).
4. D. A. Nitz, B. van Swinderen, G. Tononi, R. J. Greenspan, Electrophysiological correlates of rest and activity in *Drosophila* melanogaster. *Curr. Biol.* **12**, 1934–1940 (2002).
5. M. H. W. Yap, M. J. Grabowska, C. Rohrscheib, R. Jeans, M. Troup, A. C. Paulk, B. van Alphen, P. J. Shaw, B. van Swinderen, Oscillatory brain activity in spontaneous and induced sleep stages in flies. *Nat. Commun.* **8**, 1815 (2017).
6. B. van Alphen, M. H. W. Yap, L. Kirszenblat, B. Kottler, B. van Swinderen, A dynamic deep sleep stage in *Drosophila*. *J. Neurosci.* **33**, 6917–6927 (2013).
7. S. R. Jagannathan, T. Jeans, M. N. van de Poll, B. van Swinderen, Multivariate classification of multichannel long-term electrophysiology data identifies different sleep stages in fruit flies. *Sci. Adv.* **10**, eadj4399 (2024).
8. T. D. Wiggin, P. R. Goodwin, N. C. Donelson, C. Liu, K. Trinh, S. Sanyal, L. C. Griffith, Covert sleep-related biological processes are revealed by probabilistic analysis in *Drosophila*. *Proc. Natl. Acad. Sci. U.S.A.* **117**, 10024–10034 (2020).
9. C. B. Saper, P. M. Fuller, Wake-sleep circuitry: An overview. *Curr. Opin. Neurobiol.* **44**, 186–192 (2017).
10. A. A. Borbely, S. Daan, A. Wirz-Justice, T. Deboer, The two-process model of sleep regulation: A reappraisal. *J. Sleep Res.* **25**, 131–143 (2016).
11. N. Reinhard, F. K. Schubert, E. Bertolini, N. Hagedorn, G. Manoli, M. Sekiguchi, T. Yoshii, D. Rieger, C. Helfrich-Förster, The neuronal circuit of the dorsal circadian clock neurons in *Drosophila* melanogaster. *Front. Physiol.* **13**, 886432 (2022).
12. O. T. Shafer, A. C. Keene, The regulation of *Drosophila* sleep. *Curr. Biol.* **31**, R38–R49 (2021).
13. G. Artiushin, A. Sehgal, The *Drosophila* circuitry of sleep-wake regulation. *Curr. Opin. Neurobiol.* **44**, 243–250 (2017).
14. L. A. L. Tainton-Heap, L. C. Kirszenblat, E. T. Notaras, M. J. Grabowska, R. Jeans, K. Feng, P. J. Shaw, B. van Swinderen, A paradoxical kind of sleep in *Drosophila* melanogaster. *Curr. Biol.* **31**, 578–590.e6 (2021).
15. B. F. Fosque, Y. Sun, H. Dana, C. T. Yang, T. Ohyama, M. R. Tadross, R. Patel, M. Zlatic, D. S. Kim, M. B. Ahrens, V. Jayaraman, L. L. Looger, E. R. Schreiter, Neural circuits. Labeling of active neural circuits in vivo with designed calcium integrators. *Science* **347**, 755–760 (2015).
16. B. Moeyaert, G. Holt, R. Madangopal, A. Perez-Alvarez, B. C. Fearey, N. F. Trojanowski, J. Ledderose, T. A. Zolnik, A. das, D. Patel, T. A. Brown, R. N. S. Sachdev, B. J. Eickholt, M. E. Larkum, G. G. Turrigiano, H. Dana, C. E. Gee, T. G. Oertner, B. T. Hope, E. R. Schreiter, Improved methods for marking active neuron populations. *Nat. Commun.* **9**, 4440 (2018).
17. K. A. Edwards, M. B. Hoppa, G. Bosco, The photoconvertible fluorescent probe, CaMPARI, labels active neurons in freely-moving intact adult fruit flies. *Front. Neural Circuits* **14**, 22 (2020).
18. A. A. Bohra, B. R. Kallman, H. Reichert, K. VijayRaghavan, Identification of a single pair of interneurons for bitter taste processing in the *Drosophila* brain. *Curr. Biol.* **28**, 847–858.e3 (2018).
19. K. J. Weaver, S. Raju, R. A. Rucker, T. Chakraborty, R. A. Holt, S. D. Pletcher, Behavioral dissection of hunger states in *Drosophila*. *eLife* **12**, (2023).
20. S. Liu, Q. Liu, M. Tabuchi, M. N. Wu, Sleep drive is encoded by neural plastic changes in a dedicated circuit. *Cell* **165**, 1347–1360 (2016).
21. K. Pfeiffer, U. Homberg, Organization and functional roles of the central complex in the insect brain. *Annu. Rev. Entomol.* **59**, 165–184 (2014).
22. D. B. Turner-Evans, V. Jayaraman, The insect central complex. *Curr. Biol.* **26**, R453–R457 (2016).
23. T. Wolff, N. A. Iyer, G. M. Rubin, Neuroarchitecture and neuroanatomy of the *Drosophila* central complex: A GAL4-based dissection of protocerebral bridge neurons and circuits. *J. Comp. Neurol.* **523**, 997–1037 (2015).

24. T. Wolff, G. M. Rubin, Neuroarchitecture of the *Drosophila* central complex: A catalog of nodulus and asymmetrical body neurons and a revision of the protocerebral bridge catalog. *J. Comp. Neurol.* **526**, 2585–2611 (2018).
25. B. K. Hulse, H. Haberkern, R. Franconville, D. Turner-Evans, S. Y. Takemura, T. Wolff, M. Noorman, M. Dreher, C. Dan, R. Parekh, A. M. Hermundstad, G. M. Rubin, V. Jayaraman, A connectome of the *Drosophila* central complex reveals network motifs suitable for flexible navigation and context-dependent action selection. *eLife* **10**, e66039 (2021).
26. J. M. Donlea, M. S. Thimgan, Y. Suzuki, L. Gottschalk, P. J. Shaw, Inducing sleep by remote control facilitates memory consolidation in *Drosophila*. *Science* **332**, 1571–1576 (2011).
27. W. Yan, H. Lin, J. Yu, T. D. Wiggin, L. Wu, Z. Meng, C. Liu, L. C. Griffith, Subtype-specific roles of ellipsoid body ring neurons in sleep regulation in *Drosophila*. *J. Neurosci.* **43**, 764–786 (2023).
28. M. C. W. Ho, M. Tabuchi, X. Xie, M. P. Brown, S. Luu, S. Wang, A. L. Kolodkin, S. Liu, M. N. Wu, Sleep need-dependent changes in functional connectivity facilitate transmission of homeostatic sleep drive. *Curr. Biol.* **32**, 4957–4966.e5 (2022).
29. T. Porkka-Heiskanen, Sleep homeostasis. *Curr. Opin. Neurobiol.* **23**, 799–805 (2013).
30. C. Y. Lin, C. C. Chuang, T. E. Hua, C. C. Chen, B. J. Dickson, R. J. Greenspan, A. S. Chiang, A comprehensive wiring diagram of the protocerebral bridge for visual information processing in the *Drosophila* brain. *Cell Rep.* **3**, 1739–1753 (2013).
31. H. Luan, N. C. Peabody, C. R. Vinson, B. H. White, Refined spatial manipulation of neuronal function by combinatorial restriction of transgene expression. *Neuron* **52**, 425–436 (2006).
32. T. Kitamoto, Conditional modification of behavior in *Drosophila* by targeted expression of a temperature-sensitive shibire allele in defined neurons. *J. Neurobiol.* **47**, 81–92 (2001).
33. L. K. Scheffer, C. S. Xu, M. Januszewski, Z. Lu, S. Y. Takemura, K. J. Hayworth, G. B. Huang, K. Shinomiya, J. Maitlin-Shepard, S. Berg, J. Clements, P. M. Hubbard, W. T. Katz, L. Umayam, T. Zhao, D. Ackerman, T. Blakely, J. Bogovic, T. Dolafi, D. Kainmueller, T. Kawase, K. A. Khairy, L. Leavitt, P. H. Li, L. Lindsey, N. Neubarth, D. J. Olbris, H. Otsuna, E. T. Trautman, M. Ito, A. S. Bates, J. Goldammer, T. Wolff, R. Svirskas, P. Schlegel, E. Neace, C. J. Knecht, C. X. Alvarado, D. A. Bailey, S. Ballinger, J. A. Borycz, B. S. Canino, N. Cheatham, M. Cook, M. Dreher, O. Duclos, B. Eubanks, K. Fairbanks, S. Finley, N. Forknall, A. Francis, G. P. Hopkins, E. M. Joyce, S. J. Kim, N. A. Kirk, J. Kovalyak, S. A. Lauchie, A. Lohff, C. Maldonado, E. A. Manley, S. McLin, C. Mooney, M. Ndama, O. Ogundeyi, N. Okeoma, C. Ordish, N. Padilla, C. M. Patrick, T. Paterson, E. E. Phillips, E. M. Phillips, N. Rampally, C. Ribeiro, M. K. Robertson, J. T. Rymer, S. M. Ryan, M. Sammons, A. K. Scott, A. L. Scott, A. Shinomiya, C. Smith, K. Smith, N. L. Smith, M. A. Sobeski, A. Suleiman, J. Swift, S. Takemura, I. Talebi, D. Tarnogorska, E. Tenshaw, T. Tokhi, J. J. Walsh, T. Yang, J. A. Horne, F. Li, R. Parekh, P. K. Rivlin, V. Jayaraman, M. Costa, G. S. X. E. Jefferis, K. Ito, S. Saalfeld, R. George, I. A. Meinertzhagen, G. M. Rubin, H. F. Hess, V. Jain, S. M. Plaza, A connectome and analysis of the adult *Drosophila* central brain. *eLife* **9**, e57443 (2020).
34. S. M. Plaza, J. Clements, T. Dolafi, L. Umayam, N. N. Neubarth, L. K. Scheffer, S. Berg, neuPrint: An open access tool for EM connectomics. *Front. Neuroinform.* **16**, 896292 (2022).
35. L. J. J. Nicolai, A. Ramaekers, T. Raemaekers, A. Drozdzecki, A. S. Mauss, J. Yan, M. Landgraf, W. Annaert, B. A. Hassan, Genetically encoded dendritic marker sheds light on neuronal connectivity in *Drosophila*. *Proc. Natl. Acad. Sci. U.S.A.* **107**, 20553–20558 (2010).
36. Y. Q. Zhang, C. K. Rodesch, K. Broadie, Living synaptic vesicle marker: Synaptotagmin-GFP. *Genesis* **34**, 142–145 (2002).
37. T. Lee, L. Luo, Mosaic analysis with a repressible cell marker for studies of gene function in neuronal morphogenesis. *Neuron* **22**, 451–461 (1999).
38. J. Klarenbeek, J. Goedhart, A. van Batenburg, D. Groenewald, K. Jalink, Fourth-generation epac-based FRET sensors for cAMP feature exceptional brightness, photostability and dynamic range: Characterization of dedicated sensors for FLIM, for ratiometry and with high affinity. *PLOS ONE* **10**, e0122513 (2015).
39. O. T. Shafer, D. J. Kim, R. Dunbar-Yaffe, V. O. Nikolaev, M. J. Lohse, P. H. Taghert, Widespread receptivity to neuropeptide PDF throughout the neuronal circadian clock network of *Drosophila* revealed by real-time cyclic AMP imaging. *Neuron* **58**, 223–237 (2008).
40. L. M. DiPilato, X. Cheng, J. Zhang, Fluorescent indicators of cAMP and Epac activation reveal differential dynamics of cAMP signaling within discrete subcellular compartments. *Proc. Natl. Acad. Sci. U.S.A.* **101**, 16513–16518 (2004).
41. B. Ponsioen, J. Zhao, J. Riedl, F. Zwartkruis, G. van der Krogt, M. Zaccolo, W. H. Moolenaar, J. L. Bos, K. Jalink, Detecting cAMP-induced Epac activation by fluorescence resonance energy transfer: Epac as a novel cAMP indicator. *EMBO Rep.* **5**, 1176–1180 (2004).
42. J. Lu, A. H. Behbahani, L. Hamburg, E. A. Westeinde, P. M. Dawson, C. Lyu, G. Maimon, M. H. Dickinson, S. Druckmann, R. I. Wilson, Transforming representations of movement from body- to world-centric space. *Nature* **601**, 98–104 (2022).
43. M. Talay, E. B. Richman, N. J. Snell, G. G. Hartmann, J. D. Fisher, A. Sorkaç, J. F. Santoyo, C. Chou-Freed, N. Nair, M. Johnson, J. R. Szymanski, G. Barnea, Transsynaptic mapping of second-order taste neurons in flies by trans-Tango. *Neuron* **96**, 783–795.e4 (2017).
44. L. J. Macpherson, E. E. Zaharieva, P. J. Kearney, M. H. Alpert, T. Y. Lin, Z. Turan, C. H. Lee, M. Gallio, Dynamic labelling of neural connections in multiple colours by trans-synaptic fluorescence complementation. *Nat. Commun.* **6**, 10024 (2015).
45. W. Fouquet, D. Owald, C. Wichmann, S. Mertel, H. Depner, M. Dyba, S. Hallermann, R. J. Kittel, S. Eimer, S. J. Sigrist, Maturation of active zone assembly by *Drosophila* Bruchpilot. *J. Cell Biol.* **186**, 129–145 (2009).
46. R. J. Kittel, C. Wichmann, T. M. Rasse, W. Fouquet, M. Schmidt, A. Schmid, D. A. Wagh, C. Pawlu, R. R. Kellner, K. I. Willig, S. W. Hell, E. Buchner, M. Heckmann, S. J. Sigrist, Bruchpilot promotes active zone assembly, Ca^{2+} channel clustering, and vesicle release. *Science* **312**, 1051–1054 (2006).
47. D. A. Wagh, T. M. Rasse, E. Asan, A. Hofbauer, I. Schwenkert, H. Dürrbeck, S. Buchner, M. C. Dabauvalle, M. Schmidt, G. Qin, C. Wichmann, R. Kittel, S. J. Sigrist, E. Buchner, Bruchpilot, a protein with homology to ELKS/CAST, is required for structural integrity and function of synaptic active zones in *Drosophila*. *Neuron* **49**, 833–844 (2006).
48. T. Matkovic, M. Siebert, E. Knoche, H. Depner, S. Mertel, D. Owald, M. Schmidt, U. Thomas, A. Sickmann, D. Kamin, S. W. Hell, J. Bürger, C. Hollmann, T. Mielke, C. Wichmann, S. J. Sigrist, The Bruchpilot cytomatrix determines the size of the readily releasable pool of synaptic vesicles. *J. Cell Biol.* **202**, 667–683 (2013).
49. Y. Chen, O. Akin, A. Nern, C. Y. K. Tsui, M. Y. Pecot, S. L. Zipursky, Cell-type-specific labeling of synapses in vivo through synaptic tagging with recombination. *Neuron* **81**, 280–293 (2014).
50. R. Andretic, B. van Swinderen, R. J. Greenspan, Dopaminergic modulation of arousal in *Drosophila*. *Curr. Biol.* **15**, 1165–1175 (2005).
51. Y. Shang, P. Haynes, N. Pírez, K. I. Harrington, F. Guo, J. Pollack, P. Hong, L. C. Griffith, M. Rosbash, Imaging analysis of clock neurons reveals light buffers the wake-promoting effect of dopamine. *Nat. Neurosci.* **14**, 889–895 (2011).
52. K. Kume, S. Kume, S. K. Park, J. Hirsh, F. R. Jackson, Dopamine is a regulator of arousal in the fruit fly. *J. Neurosci.* **25**, 7377–7384 (2005).
53. Q. Liu, S. Liu, L. Kodama, M. R. Driscoll, M. N. Wu, Two dopaminergic neurons signal to the dorsal fan-shaped body to promote wakefulness in *Drosophila*. *Curr. Biol.* **22**, 2114–2123 (2012).
54. Z. Mao, R. L. Davis, Eight different types of dopaminergic neurons innervate the *Drosophila* mushroom body neuropil: Anatomical and physiological heterogeneity. *Front. Neural Circuits* **3**, 5 (2009).
55. H. K. Inagaki, S. Ben-Tabou de-Leon, A. M. Wong, S. Jagadish, H. Ishimoto, G. Barnea, T. Kitamoto, R. Axel, D. J. Anderson, Visualizing neuromodulation in vivo: TANGO-mapping of dopamine signaling reveals appetite control of sugar sensing. *Cell* **148**, 583–595 (2012).
56. G. Barnea, W. Strapps, G. Herrada, Y. Berman, J. Ong, B. Kloss, R. Axel, K. J. Lee, The genetic design of signaling cascades to record receptor activation. *Proc. Natl. Acad. Sci. U.S.A.* **105**, 64–69 (2008).
57. M. Schlichting, S. Richhariya, N. Herndon, D. Ma, J. Xin, W. Lenh, K. Abruzzi, M. Rosbash, Dopamine and GPCR-mediated modulation of DN1 clock neurons gates the circadian timing of sleep. *Proc. Natl. Acad. Sci. U.S.A.* **119**, e2206066119 (2022).
58. B. Chowdhury, L. Abhilash, A. Ortega, S. Liu, O. Shafer, Homeostatic control of deep sleep and molecular correlates of sleep pressure in *Drosophila*. *eLife* **12**, e91355 (2023).
59. J. M. Donlea, D. Pimentel, C. B. Talbot, A. Kempf, J. J. Omoto, V. Hartenstein, G. Miesenböck, Recurrent circuitry for balancing sleep need and sleep. *Neuron* **97**, 378–389.e4 (2018).
60. J. M. Donlea, D. Pimentel, G. Miesenböck, Neuronal machinery of sleep homeostasis in *Drosophila*. *Neuron* **81**, 860–872 (2014).
61. D. Pimentel, J. M. Donlea, C. B. Talbot, S. M. Song, A. J. F. Thurston, G. Miesenböck, Operation of a homeostatic sleep switch. *Nature* **536**, 333–337 (2016).
62. A. Kempf, S. M. Song, C. B. Talbot, G. Miesenböck, A potassium channel β-subunit couples mitochondrial electron transport to sleep. *Nature* **568**, 230–234 (2019).
63. D. Raccuglia, S. Huang, A. Ender, M. M. Heim, D. Laber, R. Suárez-Grimalt, A. Liotta, S. J. Sigrist, J. R. P. Geiger, D. Owald, Network-specific synchronization of electrical slow-wave oscillations regulates sleep drive in *Drosophila*. *Curr. Biol.* **29**, 3611–3621.e3 (2019).
64. J. D. Jones, B. L. Holder, K. R. Eiken, A. Vogt, A. I. Velarde, A. J. Elder, J. A. McEllin, S. Dissel, Regulation of sleep by cholinergic neurons located outside the central brain in *Drosophila*. *PLoS Biol.* **21**, e3002012 (2023).
65. J. De, M. Wu, V. Lambatan, Y. Hua, W. J. Joiner, Re-examining the role of the dorsal fan-shaped body in promoting sleep in *Drosophila*. *Curr. Biol.* **33**, 3660–3668.e4 (2023).
66. L. K. Satterfield, J. De, M. Wu, T. Qiu, W. J. Joiner, Inputs to the sleep homeostat originate outside the brain. *J. Neurosci.* **42**, 5695–5704 (2022).
67. J. Tomita, G. Ban, Y. S. Kato, K. Kume, Protocerebral bridge neurons that regulate sleep in *Drosophila melanogaster*. *Front. Neurosci.* **15**, 647117 (2021).
68. U. Hanesch, K. F. Fischbach, M. Heisenberg, Neuronal architecture of the central complex in *Drosophila melanogaster*. *Cell Tissue Res.* **257**, 343–366 (1989).
69. S. C. Renn, J. D. Armstrong, M. Yang, Z. Wang, X. An, K. Kaiser, P. H. Taghert, Genetic analysis of the *Drosophila* ellipsoid body neuropil: Organization and development of the central complex. *J. Neurobiol.* **41**, 189–207 (1999).
70. J. M. Young, J. D. Armstrong, Building the central complex in *Drosophila*: The generation and development of distinct neural subsets. *J. Comp. Neurol.* **518**, 1525–1541 (2010).

71. J. M. Young, J. D. Armstrong, Structure of the adult central complex in *Drosophila*: Organization of distinct neuronal subsets. *J. Comp. Neurol.* **518**, 1500–1524 (2010).
72. A. Lamaze, P. Krätschmer, K. F. Chen, S. Lowe, J. E. C. Jepson, A wake-promoting circadian output circuit in *Drosophila*. *Curr. Biol.* **28**, 3098–3105.e3 (2018).
73. F. Guo, M. Holla, M. M. Díaz, M. Rosbash, A circadian output circuit controls sleep-wake arousal in *Drosophila*. *Neuron* **100**, 624–635.e4 (2018).
74. T. Andreani, C. Rosensweig, S. Sisobhan, E. Ogunlana, W. Kath, R. Allada, Circadian programming of the ellipsoid body sleep homeostat in *Drosophila*. *eLife* **11**, e74327 (2022).
75. O. V. Alekseyenko, Y. B. Chan, R. Li, E. A. Kravitz, Single dopaminergic neurons that modulate aggression in *Drosophila*. *Proc. Natl. Acad. Sci. U.S.A.* **110**, 6151–6156 (2013).
76. J. D. Seelig, V. Jayaraman, Neural dynamics for landmark orientation and angular path integration. *Nature* **521**, 186–191 (2015).
77. S. S. Kim, H. Rouault, S. Druckmann, V. Jayaraman, Ring attractor dynamics in the *Drosophila* central brain. *Science* **356**, 849–853 (2017).
78. C. Lyu, L. F. Abbott, G. Maimon, Building an allocentric travelling direction signal via vector computation. *Nature* **601**, 92–97 (2022).
79. A. Flores-Valle, P. J. Gonçalves, J. D. Seelig, Integration of sleep homeostasis and navigation in *Drosophila*. *PLOS Comput. Biol.* **17**, e1009088 (2021).
80. C. Y. Ting, S. Gu, S. Guttikonda, T. Y. Lin, B. H. White, C. H. Lee, Focusing transgene expression in *Drosophila* by coupling Gal4 with a novel split-LexA expression system. *Genetics* **188**, 229–233 (2011).
81. B. D. Pfeiffer, T. T. B. Ngo, K. L. Hibbard, C. Murphy, A. Jenett, J. W. Truman, G. M. Rubin, Refinement of tools for targeted gene expression in *Drosophila*. *Genetics* **186**, 735–755 (2010).
82. J. Vienne, R. Spann, F. Guo, M. Rosbash, Age-related reduction of recovery sleep and arousal threshold in *Drosophila*. *Sleep* **39**, 1613–1624 (2016).
83. F. Guo, J. Yu, H. J. Jung, K. C. Abruzzi, W. Luo, L. C. Griffith, M. Rosbash, Circadian neuron feedback controls the *Drosophila* sleep–activity profile. *Nature* **536**, 292–297 (2016).
84. G. F. Gilestro, C. Cirelli, pySolo: A complete suite for sleep analysis in *Drosophila*. *Bioinformatics* **25**, 1466–1467 (2009).
85. N. C. Donelson, E. Z. Kim, J. B. Slawson, C. G. Vecsey, R. Huber, L. C. Griffith, High-resolution positional tracking for long-term analysis of *Drosophila* sleep and locomotion using the "tracker" program. *PLOS ONE* **7**, e37250 (2012).
86. M. Adel, N. Chen, Y. Zhang, M. L. Reed, C. Quasney, L. C. Griffith, Pairing-dependent plasticity in a dissected fly brain is input-specific and requires synaptic CaMKII enrichment and nighttime sleep. *J. Neurosci.* **42**, 4297–4310 (2022).
87. K. Kang, V. C. Panzano, E. C. Chang, L. Ni, A. M. Dainis, A. M. Jenkins, K. Regna, M. A. T. Muskavitch, P. A. Garrity, Modulation of TRPA1 thermal sensitivity enables sensory discrimination in *Drosophila*. *Nature* **481**, 76–80 (2012).
88. S. Hampel, R. Franconville, J. H. Simpson, A. M. Seeds, A neural command circuit for grooming movement control. *eLife* **4**, e08758 (2015).
89. B. D. Pfeiffer, J. W. Truman, G. M. Rubin, Using translational enhancers to increase transgene expression in *Drosophila*. *Proc. Natl. Acad. Sci. U.S.A.* **109**, 6626–6631 (2012).
90. Z. Yao, A. M. Macara, K. R. Lelito, T. Y. Minosyan, O. T. Shafer, Analysis of functional neuronal connectivity in the *Drosophila* brain. *J. Neurophysiol.* **108**, 684–696 (2012).
91. B. Deng, Q. Li, X. Liu, Y. Cao, B. Li, Y. Qian, R. Xu, R. Mao, E. Zhou, W. Zhang, J. Huang, Y. Rao, Chemoconnectomics: Mapping chemical transmission in *Drosophila*. *Neuron* **101**, 876–893.e4 (2019).
92. L. A. Perkins, L. Holderbaum, R. Tao, Y. Hu, R. Sopko, K. McCall, D. Yang-Zhou, I. Flockhart, R. Binari, H. S. Shim, A. Miller, A. Housden, M. Foos, S. Randkelv, C. Kelley, P. Namgyal, C. Villalta, L. P. Liu, X. Jiang, Q. Huan-Huan, X. Wang, A. Fujiyama, A. Toyoda, K. Ayers, A. Blum, B. Czech, R. Neumuller, D. Yan, A. Cavallaro, K. Hibbard, D. Hall, L. Cooley, G. J. Hannon, R. Lehmann, A. Parks, S. E. Mohr, R. Ueda, S. Kondo, J. Q. Ni, N. Perrimon, The transgenic RNAi project at Harvard Medical School: Resources and validation. *Genetics* **201**, 843–852 (2015).
93. F. Mohammad, J. C. Stewart, S. Ott, K. Chlebikova, J. Y. Chua, T. W. Koh, J. Ho, A. Claridge-Chang, Optogenetic inhibition of behavior with anion channelrhodopsins. *Nat. Methods* **14**, 271–274 (2017).

Acknowledgments: We thank all the Rosbash lab members, especially S. Richhariya, E. Nicholas Petersen, and K. Abruzzi, for the discussion and help for this work. We thank F. Guo, E. Zhou, R. Mao, L. Griffith, and P. Garrity for helpful discussions and comments on the manuscript. We thank K. Lyons for the assistance in maintaining the fly stocks. We thank M. Adel for the help in building up the setup for functional imaging. We thank C.-H. Lee for the UAS-LexADBD construct. We thank H. Dionne and G. Rubin (Janelia Research campus) for the UAS-shibire^{ts} fly line and Y. Rao for the Dop2R-LexA fly line. Stocks obtained from the Bloomington Drosophila Stock Center (NIH P40OD018537) were used in this study. **Funding:** This work was supported by Howard Hughes Medical Institute. **Author contributions:** Conceptualization: M.R. and X.D. Methodology: X.D., J.Q.L., and D.M. Investigation: X.D., J.Q.L., and D.M. Supervision: M.R. Writing—original draft: X.D. Writing—review and editing: M.R., X.D., J.Q.L., and D.M. Resources: X.D. and J.L. Project administration: M.R. Funding acquisition: M.R. Formal analysis: X.D. Data curation: X.D. Validation: X.D. Software: X.D. Visualization: X.D. All authors discussed the results and commented on the manuscript. **Competing interests:** The authors declare that they have no competing interests. **Data and materials availability:** All data needed to evaluate the conclusions in the paper are present in the paper and/or the Supplementary Materials.

Submitted 27 July 2024
Accepted 23 October 2024
Published 22 November 2024
10.1126/sciadv.ads0652
