# Supplementary material for: Four SpsP neurons are an integrating sleep regulation hub in Drosophila
Source: Sci Adv. 2024 Nov 22;10(47):eads0652. doi: 10.1126/sciadv.ads0652 (PMC11584021; doi:10.1126/sciadv.ads0652)
Supplement: Supplementary file 2 — Figs. S1 to S9 [file sciadv.ads0652_sm.pdf]

Science Advances
AAAS

Supplementary Materials for

Four SpsP neurons are an integrating sleep regulation hub in *Drosophila*

Xihuimin Dai *et al.*

Corresponding author: Michael Rosbash, rosbash@brandeis.edu

Sci. Adv. **10**, eads0652 (2024)
DOI: 10.1126/sciadv.ads0652

This PDF file includes:

Figs. S1 to S9

Supplementary Figures

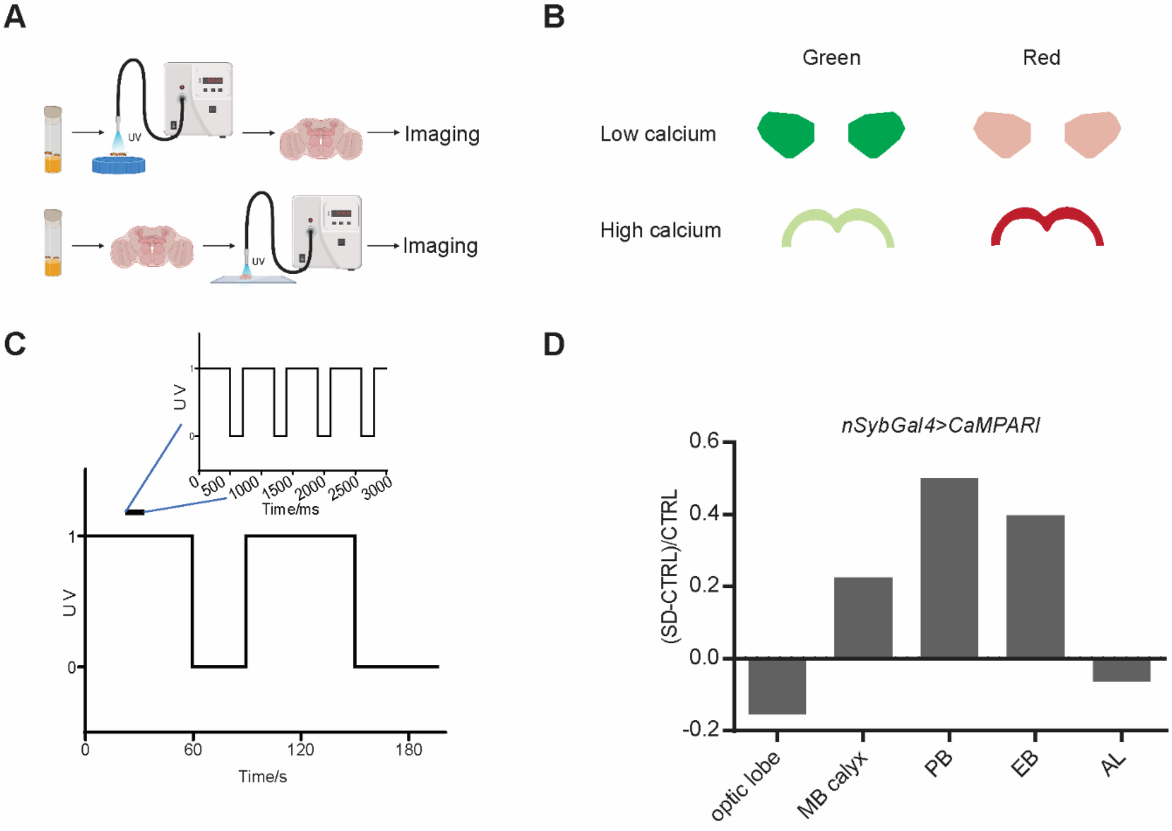

Fig. S1. Calcium activity changes in different brain regions after sleep deprivation. (A) A schematic representation of in vivo (top) and ex vivo (bottom) CaMPARI imaging. **(B)** A schematic representation of green and red signal in low calcium and high calcium brain regions at the time of UV exposure. CaMPARI converts from green to red fluorescence in the presence of UV and high concentration of calcium. Higher F_{red}/F_{green} ratio indicates a higher calcium activity. **(C)** Paradigm of UV pulsing in CaMPARI imaging. UV is pulsing with 500ms ON and 200ms OFF for 60 seconds followed by 30 seconds of break until a total duration of 150 seconds is reached. **(D)** Normalized change of CaMPARI F_{red}/F_{green} ratio between the SD group after 13hrs sleep deprivation and the CTRL group at ZT1 in different brain regions.

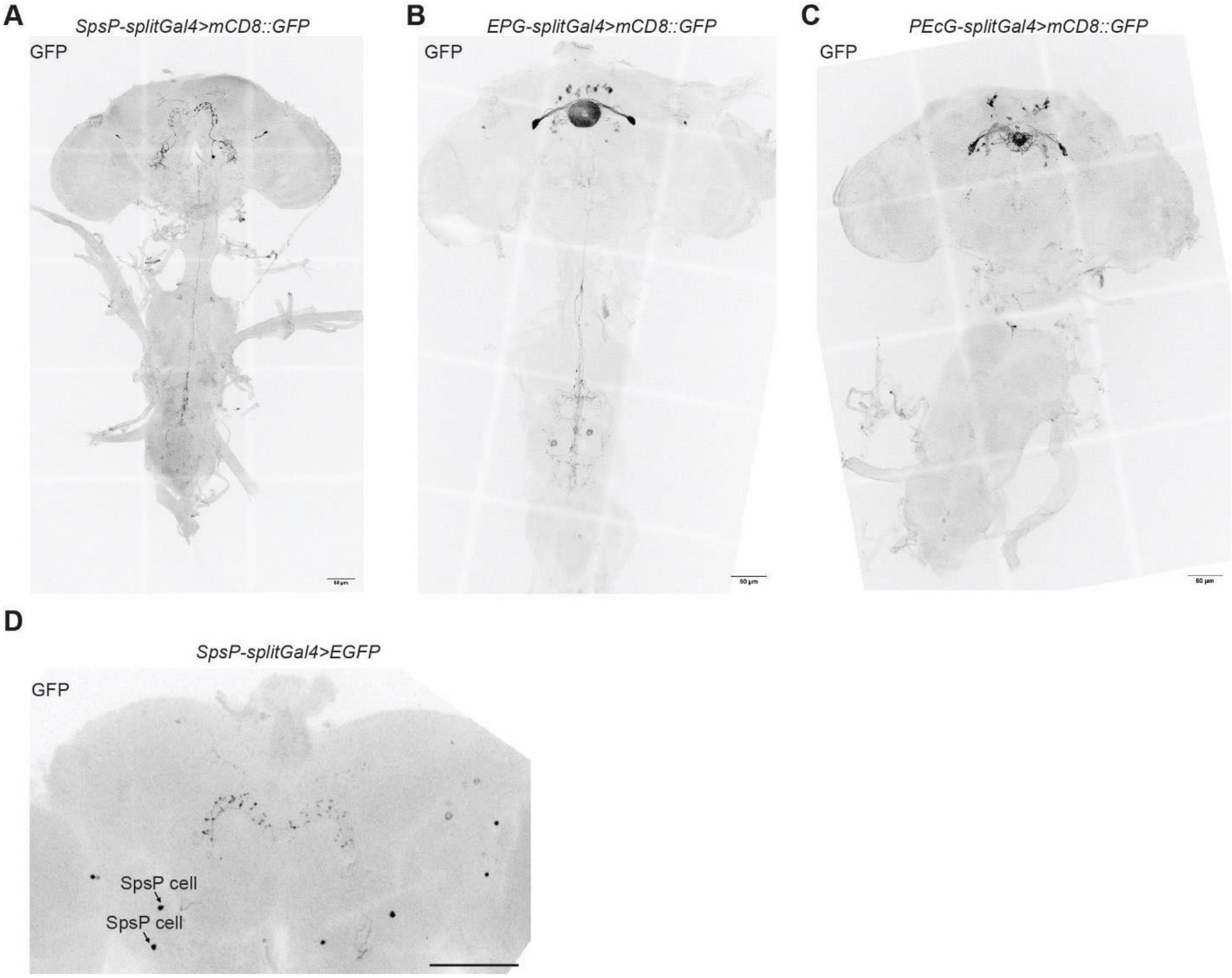

Fig. S2. Candidate split drivers show sparse and specific expression patterns. (A-C) Representative images of SpsP-splitGal4(ss52267)>mCD8::GFP **(A)**, EPG-splitGal4(ss50574)>mCD8::GFP **(B)**, and PEcG-splitGal4(ss02195)>mCD8::GFP (**C)** stained for GFP in the brain and the VNC. Scale bar: 60 µm. **(D)** Representative image of SpsP-splitGal4>EGFP stained for GFP in the brain. Arrows: Cell bodies of the SpsP cells in the left half-brain. Scale bar: 60 µm.

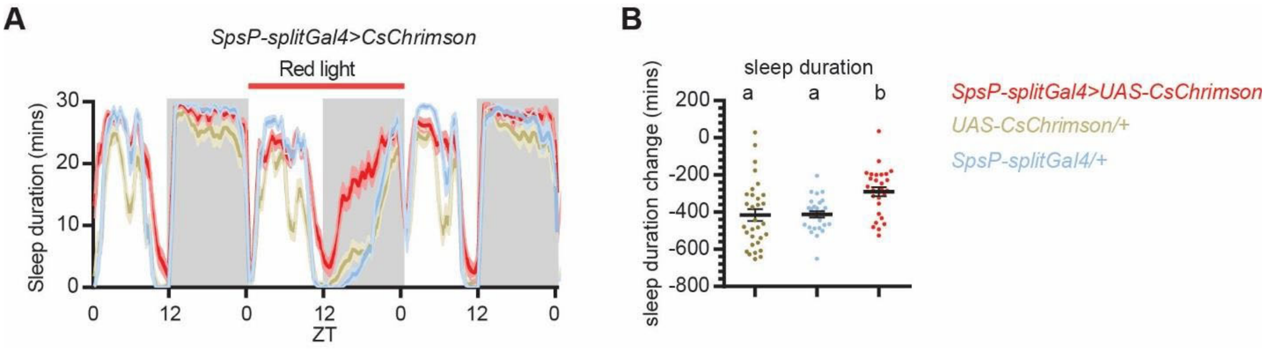

Fig. S3. Optogenetic activation of SpsP neurons promote sleep.

(A-B) Sleep profiles **(A)** and quantification of the changes of sleep duration **(B)** from ZT0-24 upon optogenetic activation of the SpsP neurons. Sleep profiles are averaged in 30 minute bins. Changes were calculated by subtracting the sleep duration of Day 1 ZT0-24 from that of Day 2. Shaded area/Error bars: S.E.M.. Red: SpsP-splitGal4>>UAS-CsChrimson (n=28); Yellow: UAS-CsChrimson/+ (n=31); Blue: SpsP-splitGal4/+ (n=29). Letters represent statistically distinct groups; $P < 0.01$, Kruskal–Wallis test followed by a post hoc Dunn's test. Male flies were used. Male flies were used.

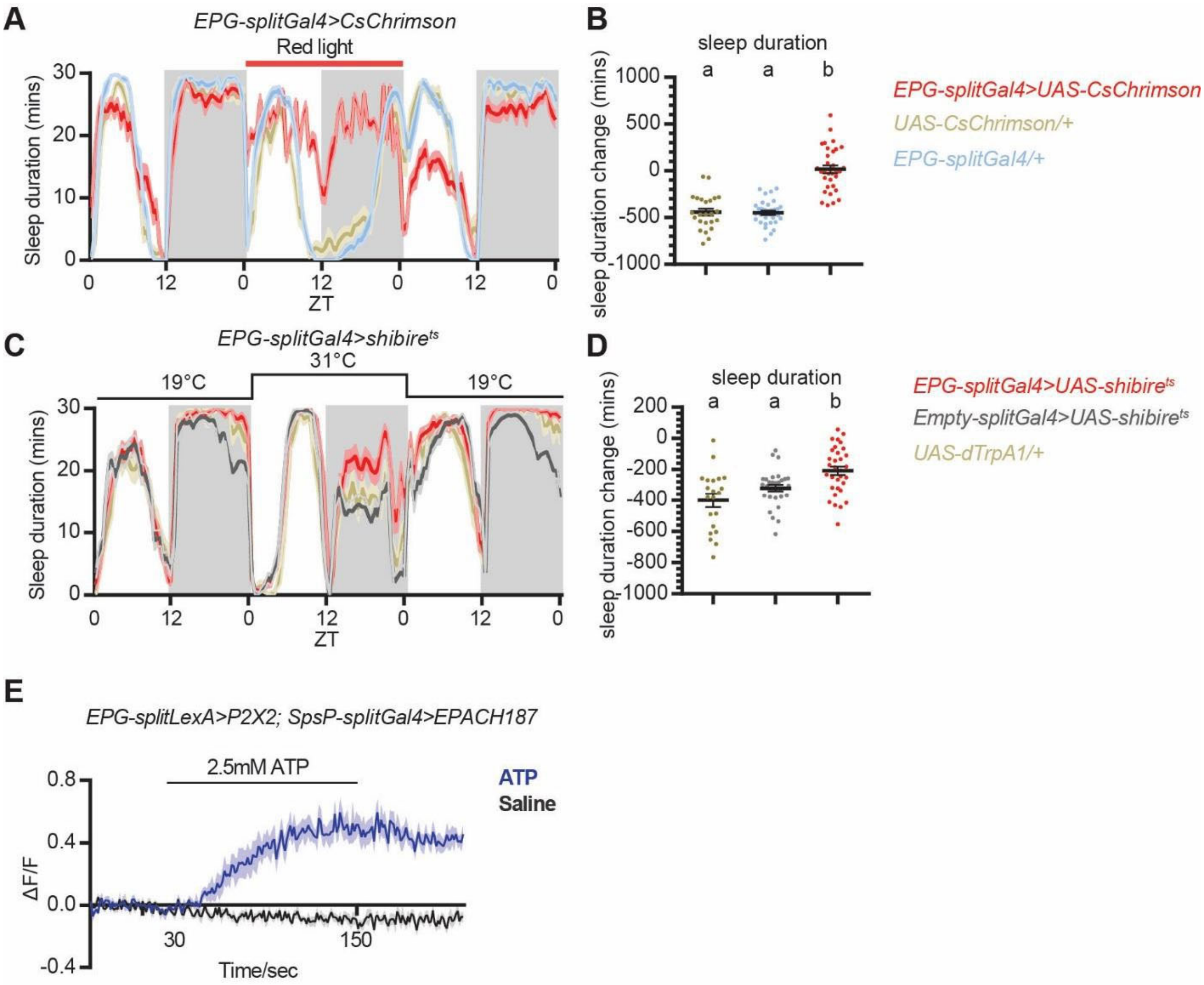

Fig. S4. Activation of EPG neurons causes increased sleep and increased ATP level in SpsP neurons, whereas inhibition of E-PG neurons causes mildly increased nighttime sleep. Sleep profiles **(A, C)** and quantification of the changes in sleep duration **(B, D)** from ZT0-24 upon optogenetic activation **(A)** or neurotransmitter release blocking **(C-D)** of the EPG neurons. Sleep profiles are averaged in 30 minute bins. Changes were calculated by subtracting the sleep duration of Day 1 ZT0-24 from that of Day 2. Shaded area/Error bars: S.E.M.. Red: EPG-splitGal4/UAS; Yellow: UAS/+; Blue: EPG-splitGal4/+; Gray: Empty-splitGal4/UAS. More than 20 male flies were used for each group. Letters represent statistically distinct groups; $P < 0.05$, Kruskal–Wallis test followed by a post hoc Dunn's test. **(E)** Average EPAC traces (ΔF/F:

inverse FRET signal) of SpsP cells in response to EPG activation. More than 7 male flies were used for each group. Error bars: S.E.M..

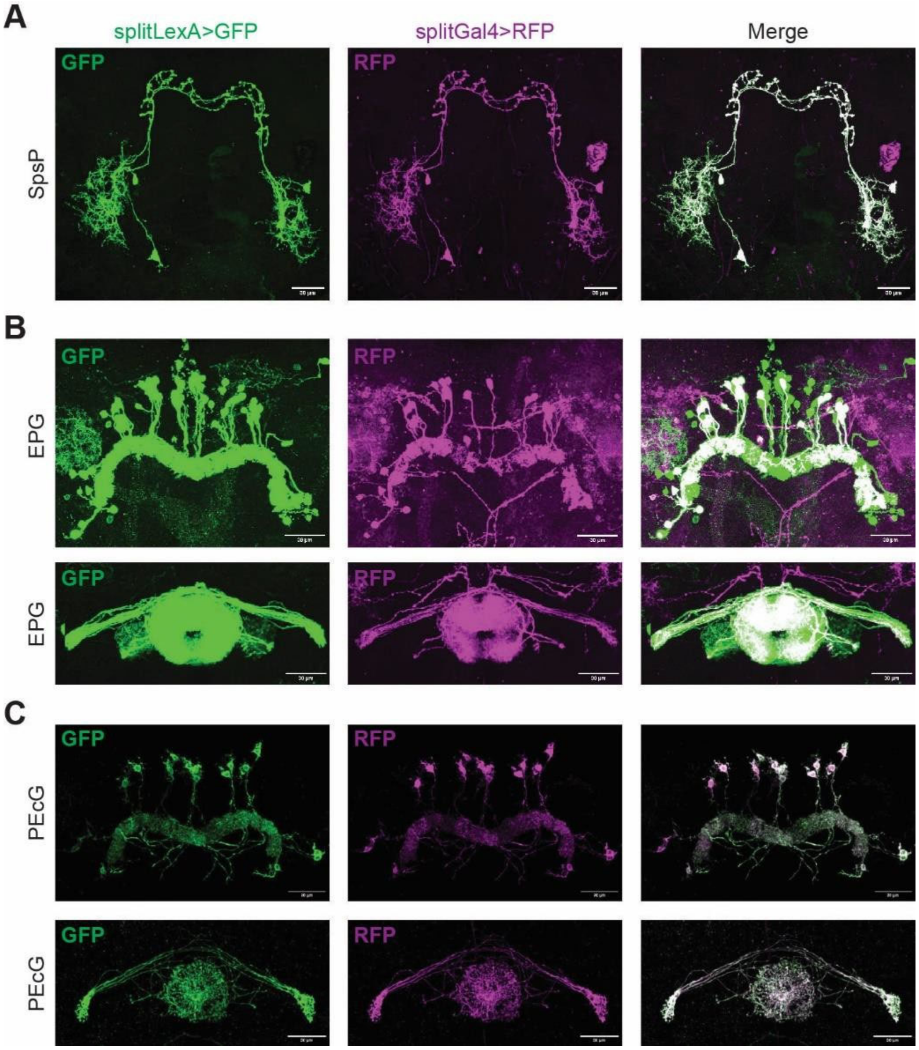

Fig. S5. The expression patterns of split-LexAs recapitulate those of corresponding split-Gal4s. Representative images of neurons labeled by split-LexA drivers (left) and split-Gal4 drivers of SpsP **(A)**, EPG **(B)**, and PEcG **(C)** stained for GFP and dsRed. Scale bar: 30 µm.

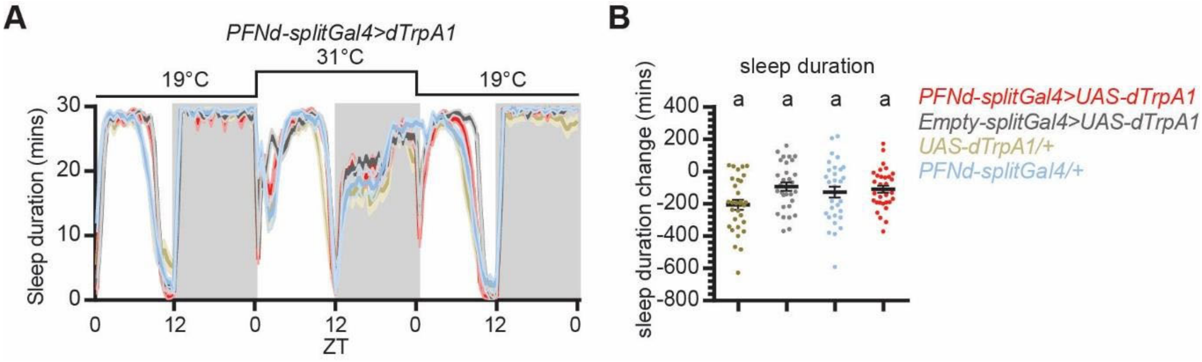

Fig. S6. Activation of the PFNd neurons has no effect in sleep duration. Sleep profiles **(A)** and quantification of the changes in sleep duration **(B)** from ZT0-24 upon thermogenetic activation of the PFNd neurons. Heatshock was from Day2 ZT0-24. Sleep profiles are averaged in 30 minute bins. Changes were calculated by subtracting the sleep duration of Day 1 ZT0-24 from that of Day 2. Shaded area/Error bars: S.E.M.. Red: PFNd-splitGal4>UAS-dTrpA1 (n=32); Gray: Empty-splitGal4/UAS-dTrpA1 (n=31); Yellow: UAS-dTrpA1/+ (n=31); Blue: PFNd-splitGal4/+ (n=31). Same letters represent statistically indistinct groups; $P > 0.05$, Kruskal–Wallis test followed by a post hoc Dunn's test. Male flies were used.

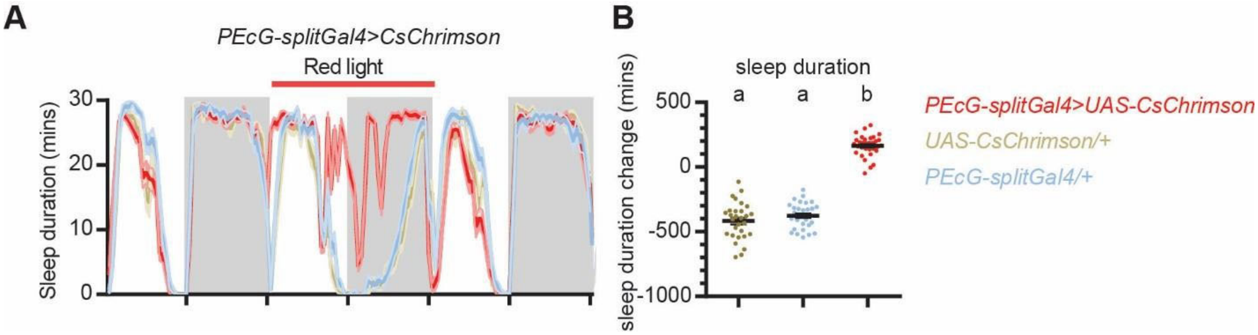

Fig. S7. Activation of the PEcG neurons causes a sleep increase. Sleep profiles **(A)** and quantification of the changes in sleep duration **(B)** of ZT0-24 upon optogenetic activation of the P-EcG neurons. Sleep profiles are averaged in 30 minute bins. Shaded area/Error bars: S.E.M.. Changes were calculated by subtracting the sleep duration of Day 1 ZT0-24 from that of Day 2. Red: P-EcG-splitGal4>UAS-CsChrimson (n=32); Yellow: UAS-CsChrimson/+ (n=29); Blue: P-EcG-splitGal4/+ (n=30). Letters represent statistically distinct groups; P < 0.0001, Kruskal–Wallis test followed by a post hoc Dunn's test.

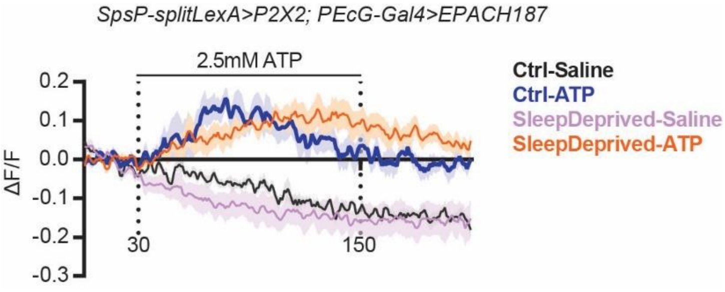

Fig. S8. Activation of the SpsP neurons causes cAMP level increase in the P-EcG neurons.

Averaged EPAC traces (ΔF/F, F: inverse FRET signal) of PEcG cells in response to ATP-induced SpsP activation. n=5 male flies for each group. Error bars: S.E.M..

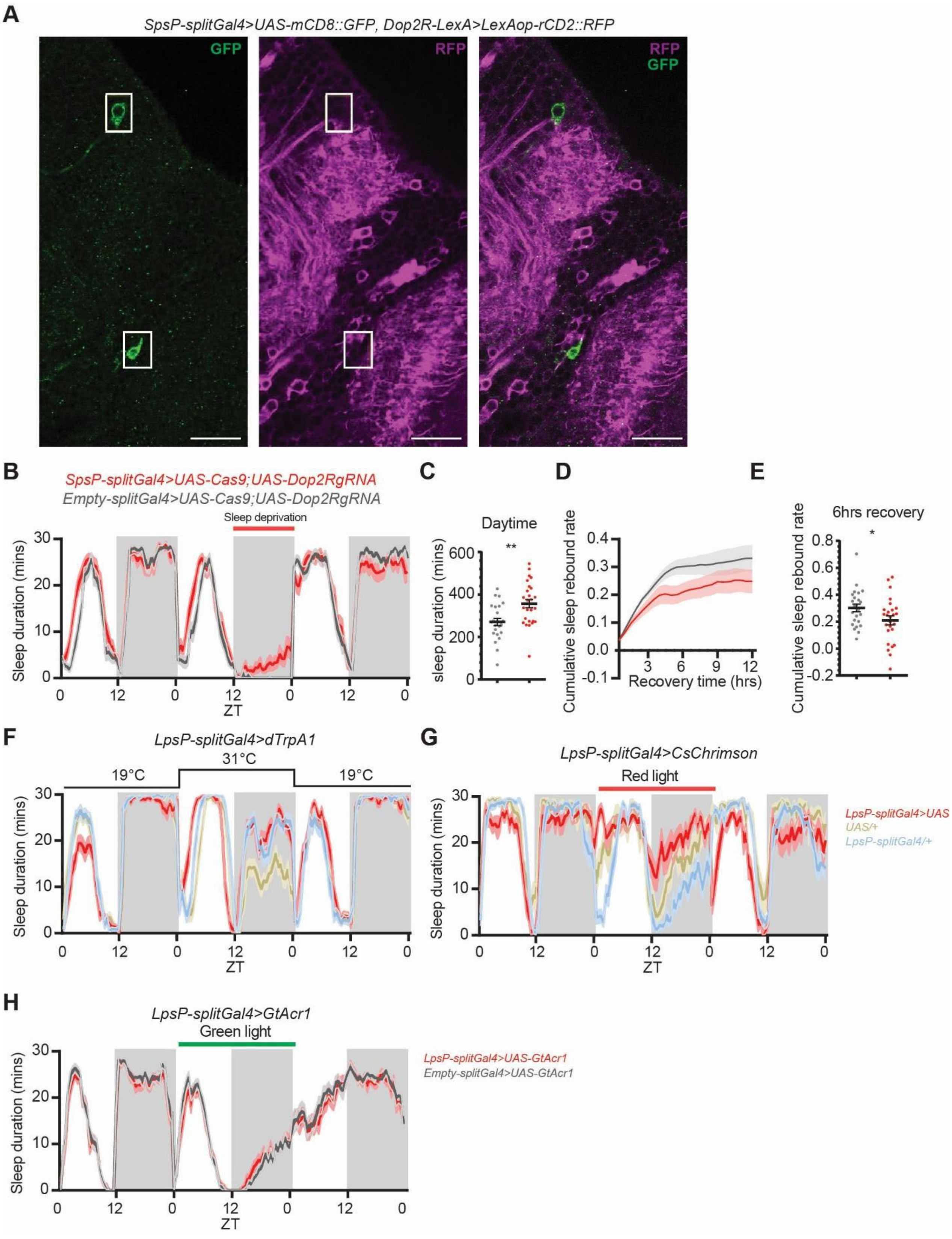

Fig. S9. SpsP neurons integrate dopaminergic signaling to regulate sleep.

(A) Representative images of the additional neurons labeled by the SpsP driver (green) and the Dop2R-expressing neurons (magenta) labeled by SpsP-splitGal4>UAS-mCD8::GFP, Dop2R-LexA>LexAop-rCD2::RFP stained for dsRed and GFP. Scale bar: 20 μm.

(B-E) Sleep profiles **(B)**, quantification of the daytime sleep duration from ZT0-12 **(C)**, cumulative sleep rebound rate curve in 12 hours **(D)**, and the quantification of cumulative sleep rebound rate after 6 hours of recovery sleep **(E)** in the experimental group and the control groups of SpsP specific Dop2R mutated flies. **(B)** and **(D)** are in 30 minute bins. Shaded area/Error bars: S.E.M.. Red: SpsP-splitGal4>UAS-Cas9; UAS-Dop2RgRNA (n=26); Gray: Empty-splitGal4>UAS-Cas9; UAS-Dop2RgRNA (n=24). *P<0.05, **P<0.01, Mann-Whitney test. Virgin female flies were used.

(F-H) Sleep profiles of thermogenetic activation **(F)**, optogenetic activation **(G)**, and optogenetic inhibition **(H)** of the dopaminergic LpsP neurons in 30 minute bins. Red: LpsP-splitGal4/UAS; Yellow: UAS/+; Blue: LpsP-splitGal4/+; Gray: Empty-splitGal4/UAS. More than 20 male flies were used for each group.
